# Associations between health behaviours, fertility and reproductive outcomes: triangulation of evidence in the Norwegian Mother, Father and Child Cohort Study (MoBa)

**DOI:** 10.1186/s12916-023-02831-9

**Published:** 2023-04-03

**Authors:** Robyn E. Wootton, Rebecca B. Lawn, Maria C. Magnus, Jorien L. Treur, Elizabeth C. Corfield, Pål R. Njølstad, Ole A. Andreassen, Deborah A. Lawlor, Marcus R. Munafò, Siri E. Håberg, George Davey Smith, Ted Reichborn-Kjennerud, Per Magnus, Alexandra Havdahl

**Affiliations:** 1 Nic Waals Institute, Lovisenberg Diaconal Hospital, Oslo, Norway; 2 MRC Integrative Epidemiology Unit, University of Bristol, Bristol, UK; 3 Population Health Sciences, Bristol Medical School, University of Bristol, Bristol, UK; 4 School of Psychological Science, University of Bristol, Bristol, UK; 5 Centre for Fertility and Health, Norwegian Institute of Public Health, Oslo, Norway; 6 Department of Epidemiology, Harvard T.H. Chan School of Public Health, Boston, MA USA; 7 Department of Psychiatry, Amsterdam UMC, University of Amsterdam, Amsterdam, The Netherlands; 8 Department of Mental Disorders, Norwegian Institute of Public Health, Oslo, Norway; 9 Center for Diabetes Research, Department of Clinical Science, University of Bergen, Bergen, Norway; 10 Children and Youth Clinic, Haukeland University Hospital, Bergen, Norway; 11 NORMENT Centre, Division of Mental Health and Addiction, Institute of Clinical Medicine, University of Oslo, Oslo University Hospital, Oslo, Norway; 12 Institute of Clinical Medicine, University of Oslo, Oslo, Norway; 13 Center for Genetic Epidemiology and Mental Health, Norwegian Institute of Public Health, Oslo, Norway

**Keywords:** Fertility, Reproductive outcomes, Alcohol, Smoking, BMI, Caffeine, Mendelian randomisation, MoBa, MBRN

## Abstract

**Background:**

Guidance to improve fertility includes reducing alcohol and caffeine consumption, achieving healthy weight-range and stopping smoking. Advice is informed by observational evidence, which is often biased by confounding.

**Methods:**

This study primarily used data from a pregnancy cohort, the Norwegian Mother, Father and Child Cohort Study. First, we conducted multivariable regression of health behaviours (alcohol and caffeine consumption, body-mass index (BMI), and smoking) on fertility outcomes (e.g. time to conception) and reproductive outcomes (e.g. age at first birth) (*n* = 84,075 females, 68,002 males), adjusting for birth year, education and attention-deficit and hyperactive-impulsive (ADHD) traits. Second, we used individual-level Mendelian randomisation (MR) to explore possible causal effects of health behaviours on fertility/reproductive outcomes (*n* = 63,376 females, 45,460 males). Finally, we performed summary-level MR for available outcomes in UK Biobank (*n* = 91,462–1,232,091) and controlled for education and ADHD liability using multivariable MR.

**Results:**

In multivariable regression analyses, higher BMI associated with fertility (longer time to conception, increased odds of infertility treatment and miscarriage), and smoking was associated with longer time to conception. In individual-level MR analyses, there was strong evidence for effects of smoking initiation and higher BMI on younger age at first birth, of higher BMI on increased time to conception, and weak evidence for effects of smoking initiation on increased time to conception. Age at first birth associations were replicated in summary-level MR analysis; however, effects attenuated using multivariable MR.

**Conclusions:**

Smoking behaviour and BMI showed the most consistent associations for increased time to conception and a younger age at first birth. Given that age at first birth and time to conception are positively correlated, this suggests that the mechanisms for reproductive outcomes are distinct to the mechanisms acting on fertility outcomes. Multivariable MR suggested that effects on age at first birth might be explained by underlying liability to ADHD and education.

**Supplementary Information:**

The online version contains supplementary material available at 10.1186/s12916-023-02831-9.

## Background

Couples struggling to conceive are often advised to engage in healthier lifestyle behaviours, for example, reducing their alcohol and caffeine consumption, achieving a healthy weight-range and quitting smoking [1–3]. Reviews of observational evidence support an association between these health behaviours and reduced fertility in females [4], with high alcohol consumption and smoking associated with reduced likelihood of conception [5–9] and high caffeine consumption and obesity associated with increased risk of miscarriage [10, 11]. Epidemiological research on fertility and reproductive outcomes often focuses on females [2, 12, 13]; however, it is important to also consider the impact of health behaviours in the partner. Meta-analyses suggest that smoking and alcohol consumption can reduce semen quality [14], and obesity in males has been associated with reduced likelihood of natural conception [15] and an increased time to conception [16]. In the current study, we explored a range of outcomes to get a more complete picture of the effects of health behaviours. Time to conception, use of infertility treatment and miscarriage are closely related to a couple’s fertility, and herein we refer to them as “fertility” outcomes. We also explored downstream “reproductive” outcomes (age at first birth and number of children), which are interconnected with fertility, but are further independently influenced by numerous societal, behavioural, and psychological factors.

The majority of evidence to date assessing the impact of health behaviours on fertility and reproductive outcomes is observational, and most studies do not adequately control for confounding [17]. For example, health behaviours often co-occur with other health behaviours which might instead affect fertility/reproductive outcomes (e.g. diet, physical activity, sleep). Furthermore, health behaviours and reproductive outcomes such as age at first birth and number of children share common predictors including low educational attainment and liability to inattention and hyperactive-impulsive behaviour (traits of attention deficit hyperactivity disorder; ADHD) [18–20]. In addition to confounding, it is necessary to account for reverse causation because the stress of being unable to conceive might cause couples to engage in unhealthy behaviours. We extended previous epidemiological studies using Mendelian randomisation (MR), which can reduce bias from residual confounding and reverse causation. MR uses genetic variants (single nucleotide polymorphisms; SNPs) to estimate the causal effect of an exposure on an outcome [21]. Evidence from MR can be triangulated with existing evidence to inform fertility guidance. More evidence is useful for helping couples successfully conceive as well as removing any unnecessary stress or guilt around unrelated lifestyle factors. The objective of the current study was to explore the associations between health behaviours on fertility and reproductive outcomes in both males and females using an MR approach. We predominantly used the Norwegian Mother, Father and Child (MoBa) pregnancy cohort, containing around 95,000 mothers and 75,000 partners.

## Methods

### Sample

MoBa is a prospective population-based pregnancy cohort study conducted by the Norwegian Institute of Public Health. Pregnant women were recruited from all over Norway from 1999 to 2008 [22, 23]. Women consented to participate in 41% of the pregnancies. The cohort includes 114,500 children, 95,200 mothers, and 75,200 partners. The current study was based on version 12 of the quality-assured data files released for research in January 2019. Questionnaires were completed across multiple time points during pregnancy and after birth. The present study used measures from the first questionnaires received between 13 and 17 weeks gestation (herein referred to as the 15-week questionnaire). The questions related to previous pregnancies, medical history and medication use, occupation, exposures in the workplace and home, lifestyle habits and mental health. In addition to questionnaire data, information on parental characteristics and pregnancy outcomes of the index pregnancies were available through data linkage to the Medical Birth Registry Norway (MBRN). MBRN also provided data on age at first birth and total number of children up to 2018, including (but not limited to) the MoBa index pregnancy. After restricting to those with available exposure and outcome data, a maximum of 84,075 females and 68,002 males were included in multivariable regression analysis. After further restricting to those individuals with quality-controlled genotype data (see details below), a maximum of 63,376 females and 45,460 males were available for individual-level MR analysis. More details of participant exclusion are given in Additional file 1: Fig. S1.

### Health behaviours exposure data

For all health behaviours, we used data from the 15-week questionnaire in pregnancy. Females were asked to report their behaviour 3 months prior to the index pregnancy. Males were asked to report their behaviour 6 months prior to the index pregnancy.

#### Alcohol consumption

Frequency of alcohol consumption was self-reported on a 7-point scale (never, less than once a month, 1–3 times a month, once per week, 2–3 times per week, 4–5 times per week, 6–7 times per week). Binge drinking was self-reported (“How often did you drink 5 units or more on one occasion?”) on a 5-point scale (never, less than once a month, 1–3 times a month, once a week, several times per week).

#### Caffeine consumption

Caffeine consumption in females was calculated from self-reported daily beverage consumption, where one cup contained 125 ml. Caffeine (mg) weights per cup were taken from a previous study of caffeine consumption in MoBa [24]. We excluded outliers if they consumed more than 3.5 l of any particular drink in a day (28 cups) or if their total caffeine consumption per day was greater than 1000 mg [25]. Values were log transformed to adjust for skewness. In males, beverage consumption was instead measured categorically over a typical week. Responses were on a 5-point scale (seldom/never, 1–6 cups a week, 1 cup a day, 2–3 cups a day, 4 + cups a day). Unlike the questionnaire administered to females, males were not directed as to the volume of the cup; therefore, we followed previous calculations [24] and assumed that a cup was 125 ml for coffee (apart from espresso where we assumed a standard single is 30 ml) and 250 ml for tea or fizzy drink. Consumption was weighted by caffeine (mg) [24] and divided by 7 to estimate mg per day.

#### Smoking behaviour

Smoking initiation was self-reported: “Have you ever smoked?” where yes was classed as ever smoking and no was classed as never smoking. Smoking heaviness was self-reported amongst current smokers as the average number of cigarettes smoked per day prior to pregnancy. We excluded current smokers who reported smoking no cigarettes.

#### Body mass index (BMI)

Height and weight were self-reported. We used pre-pregnancy values for females and current (15 weeks) values for males. Females were also asked to report their partner’s height and weight. These reports were highly correlated with partners’ self-report (*r* = 0.98 for height and *r* = 0.95 for weight). Therefore, we used the female’s report of their partner’s height and weight when the male’s own report was unavailable. We excluded outliers for females at height < 117 or > 196 cm and weight < 38 or > 150 kg and for males at height < 136 or > 220 cm and weight < 50 or > 200 kg as done previously in MoBa [16]. From these height and weight measures, we calculated BMI as weight (kg)/height (m^2^). We note BMI is not a health behaviour itself but is a biomarker which can crudely proxy for healthy behaviours.

### Fertility outcome data

All fertility outcomes (time to conception, use of infertility treatment and miscarriage) were self-reported in the 15-week questionnaire by the females.

#### Time to conception

This is based upon the female self-report from the 15-week questionnaire referring to the index pregnancy. If females reported planning their pregnancy, they were asked: “For how many months did you have regular intercourse without contraception before you became pregnant?”. Options were less than 1 month, 1–2 months or 3 + months. If it took more than 3 months, then they were asked to state the number of months. We combined anyone taking 12 or more months to conceive into one group to reduce skewness and treated as a continuous variable. We have used this female-reported variable as an outcome in both females and males under the assumption that couples were conceiving together. In our primary analysis, if the couple were not trying to conceive, then they were set to missing. However, given differences between couples who planned pregnancy and those who did not, we also conducted a sensitivity analysis where non-planners were included and assigned the median time to conception from the planning group (2 months) (Additional file 1: Note S1). We also conducted a sensitivity analysis using dichotomised variables as time to conception was not measured continuously (Additional file 1: Note S2).

#### Infertility treatment

Females self-reported in the 15-week questionnaire: “Have you ever been treated for infertility?”. Responses were binary yes or no. We did not use this variable as an outcome in the males, as this question did not specify whether the infertility treatment was for the index pregnancy.

#### Miscarriage

Females self-reported (in the 15-week questionnaire) ever having had a miscarriage, defined as any of their previous pregnancies ending in spontaneous abortion at or before the 20th week of pregnancy.

### Reproductive outcome data

Reproductive outcomes (age at first birth and total number of children) were obtained from the Medical Birth Registry of Norway (MBRN) for both females and males (last updated November 2018).

#### Age at first birth

Age at the time of first recorded child’s delivery was obtained from MBRN (not limited to births recorded in MoBa).

#### Number of children

The total number of children born up to November 2018 was obtained from MBRN (including but not limited to births recorded in MoBa). Totals of more than 6 children were grouped into a 6 + category to adjust for skewness.

As a secondary outcome, we also explored the impact of health behaviours on frequency of sexual intercourse (see Additional file 1: Note S3).

### Genotype data

Blood samples were obtained from MoBa parents during pregnancy [26]. For the current study, we used the most recent release of quality controlled genotype data available for the MoBa sample [27]. For an overview of quality control (QC) steps and exclusions, see Additional file 1: Fig S1. Further details of the genotyping and QC procedures are available elsewhere [27]. After QC and relatedness checks, the remaining samples contained 71,116 females and 50,204 males.

### Genetic score construction

For individual-level MR, our genetic instruments were individual-level genetic scores constructed in the PRSice package [28] using genome-wide significant variants. SNPs were clumped to ensure independence (*r*^2^ < 0.01, clumping window < 1000 kilobases) and weighted by effect sizes from discovery GWAS detailed below (selected for being the largest samples using individuals of European ancestry and not containing the MoBa cohort). Prior to analysis, we checked that each genetic score explained significant variance in the exposure. This is presented in Additional file 1: Table S1 along with the number of SNPs passing QC in the MoBa cohort.

### GWAS summary statistics for health behaviours

The following GWAS summary statistics were used to construct genetic scores for individual-level MR, and individual SNP effects sizes were used in summary-level MR.

#### Alcohol consumption

We used two genetic instruments for alcohol consumption: (1) alcohol consumption frequency and (2) binge drinking. Alcohol consumption frequency was measured as average number of drinks per week aggregated across types of alcoholic beverage. The GWAS identified 99 conditionally independent genome-wide significant SNPs in a sample of 941,280 individuals, explaining 2.5% of the variance [29]. Binge drinking in the UK Biobank was defined as “How often do you have six or more drinks on one occasion?”, where a drink was defined as a unit of alcohol. The GWAS was conducted by the Neale Lab (http://www.nealelab.is/uk-biobank—round 2, August 2018), and variance explained in an independent sample was not reported. After restricting to independent variants, 4 genome-wide significant SNPs remained.

#### Caffeine consumption

Caffeine consumption was measured as number of cups of coffee per day. The GWAS identified 6 independent genome-wide significant SNPs in a sample of 91,462 coffee drinkers of European ancestry [30]. Genome-wide significant SNPs explained 1.3% of the variance in coffee consumption.

#### Smoking behaviour

We used two genetic instruments for smoking behaviour: (1) smoking initiation and (2) smoking heaviness. Smoking initiation was defined as ever v. never regularly smoking (more than 100 cigarettes ever or having ever been a daily smoker). The GWAS of smoking initiation identified 378 conditionally independent genome-wide significant SNPs, in a sample of 1,232,091 individuals, which explained 4% of the variance [29]. Smoking heaviness was defined as the average number of cigarettes smoked per day. The GWAS of smoking heaviness identified 55 conditionally independent genome-wide significant SNPs in a sample of 337,334 ever smokers, which explained 4% of the variance [29]. We also conducted a single-SNP analysis of rs16969968 genotype from the CHRNA5 gene, known to reduce nicotine aversion and consequently increase cigarettes smoked per day [31] (see Additional file 1: Note S4 [32–37]).

#### Body mass index (BMI)

The most recent GWAS of adult BMI identified 941 independent SNPs at *p* < 1 × 10^−8^ in a sample of 681,275 individuals of European ancestry, which explain 6% of the variance in BMI [38].

### Statistical analysis

All analyses were conducted in R version 4.0.3 [39] and performed separately for females and males. We corrected for multiple testing using a Bonferroni correction of 0.05/48 tests (6 exposures and 8 outcomes) which resulted in an adjusted *p*-value of *p* < 0.001.

#### Stage 1. Multivariable regression analyses

We first explored the associations between each of the fertility and reproductive outcomes using correlation for continuous traits, chi-squared tests for binary traits and independent *t*-tests when one trait was continuous and the other binary. Second, to test the association with health behaviours, we conducted linear regressions for each continuous outcome and logistic regressions for each binary outcome. Results are presented unadjusted and adjusted for common confounders: birth year, educational attainment (at around 15-weeks pregnancy) and for ADHD traits as a sensitivity analysis to proxy for ADHD liability. ADHD traits were measured when the index child was age 3 years using the Adult ADHD Self-Report Scale [40].

#### Stage 2. Individual-level Mendelian randomisation

MR can be implemented as an instrumental variable analysis using genetic variants to proxy for an exposure. It can be used to estimate a causal effect of the (health behaviour) exposures on the (fertility and reproductive) outcomes providing certain assumptions are satisfied [21, 41]. The three core assumptions for valid causal inference are as follows: (1) relevance—the genetic instrument must be robustly associated with the exposure, (2) independence—there should be no confounding between the genetic instrument and outcome, and (3) exclusion-restriction—the genetic instrument must only be associated with the outcome via the exposure. Additionally, for results to generalise to other populations, there must be no effect modification, such that the causal effect of exposure on outcome is unrelated to the genetic instrument in both the subpopulations of exposed and unexposed individuals (the homogeneity assumption) [41]. Given the implausibility of this assumption, we instead aim to calculate the local average treatment effect (satisfying the monotonicity assumption), such that there are no “defiers”—individuals whose exposure is opposite to their genetic predisposition [41].

Our individual-level MR analysis used individual-level genetic scores (with weights from external independent GWAS) in an instrumental variable regression controlling for age, genotyping batch, imputation batch and top 10 principal components of population structure. We used two-stage least squares instrumental variable analyses to estimate the causal effects [42]. Specifically, the standardised genetic scores were first regressed on the exposure, and then predicted values were regressed onto the outcome. For continuous outcomes, betas are the mean difference in the outcome per unit increase in the genetically predicted exposure. For binary outcomes, estimates approximate the risk difference of the outcome per unit increase in the genetically predicted exposure. When smoking initiation is the exposure, one unit is one standard deviation (SD) increase in the probability of initiating smoking. Analyses were performed using the *ivreg* command from the Applied Econometrics with R (*AER)* package. Power calculations for individual-level MR are presented in Additional file 1: Note S5 [43, 44].

##### Sensitivity analyses

We checked for evidence of assortative mating by using the female’s genetic score to predict their partner’s health behaviours and vice versa and by estimating the correlation between their genetic scores. We checked for evidence of possible pleiotropy by testing whether each of the genetic scores predicted any known confounders of the exposure-outcome association (e.g. other health behaviours, income, age) and compared these estimates with the estimated association between observed exposures on confounders. Pleiotropy occurs when one genetic variant influences multiple phenotypes. If these other phenotypes are not on the causal pathway from exposure to outcome (horizontal pleiotropy) then the independence and exclusion-restriction assumptions could be violated, and genetic variants are invalid instruments. Where there was evidence for a causal effect in the individual-level MR analysis (after correction for multiple testing), we followed up with additional summary-level MR sensitivity analyses (MR Egger [45], weighted median [46] and weighted mode [47]) which make different assumptions about the nature of pleiotropy. A consistent direction of effect across the different MR sensitivity analyses gives us more confidence that the effects are not due to pleiotropy. We also calculated the MR Egger intercept. If the intercept is significantly different from zero, then this suggests significant directional horizontal pleiotropy may be biasing the estimate. To conduct these summary-level sensitivity MR analyses, we generated SNP-outcome association results for the MoBa cohort, adjusting for age, genotyping batch, imputation batch and 10 PCs. To test for possible reverse causation of individuals having their second child, we compared the individual-level MR results of the full sample, with those in a sample restricted to couples for whom this was their first pregnancy (Additional file 1: Note S6 [48]).

#### Stage 3. Summary-level mendelian randomisation

The second MR method used was summary-level MR (i.e. two-sample MR) which uses summary statistics from published GWAS [49]. Here, we do not have an effect estimate for each individual but instead an effect size for each SNP from the discovery GWAS and an effect size for that SNP in an outcome GWAS. The ratio of these two effect sizes can be meta-analysed across multiple SNPs to give an estimate of the causal effect. This is our primary analysis known as the inverse-variance weighted estimate. As sensitivity analyses, again we conducted MR Egger [45], weighted median [46] and weighted mode [47], which each make orthogonal assumptions about the nature of pleiotropy.

Independent summary GWAS data was available for three of the outcomes: age at first birth (*N* = 170,498), number of children (*N* = 333,628) and number of miscarriages (*N* = 78,700), self-reported in the UK Biobank [50]. We used outcome GWAS from the UK Biobank to prevent sample overlap with our health behaviour exposure GWAS. For age at first birth and number of miscarriages, we obtained GWAS summary statistics from the MRC IEU Open GWAS project [51]. The GWAS for age at first birth used the self-reported question “How old were you when you had your first child?” (field 2754) asked only to females who had previously indicated that they had given birth to at least one child. The GWAS for number of miscarriages used the item “How many spontaneous miscarriages have you had?” (field 3839) which was only asked to females who had previously indicated that they had ever had a miscarriage, abortion or stillbirth (field 2774). Finally, we used a GWAS for number of children, constructed by combining UK Biobank items “How many children have you fathered?” (field 2405) in males and “How many children have you given birth to? (Please include live births only)” (field 2734) in females [52]. Age at first birth and number of miscarriages GWASs are in standard deviation (SD) units and units for number of children are number of children.

For each of the exposures, we used the same health behaviour GWAS as for the individual-level MR, with the exception of BMI. Here we used an earlier GWAS [53] that did not contain the UK Biobank to avoid sample overlap which can bias MR estimates [54]. For smoking initiation and alcohol consumption, we used SNP-exposure estimates from summary statistics with the UK Biobank and 23andMe removed. Smoking heaviness could not be used as an exposure because the outcome GWASs could not be stratified on smoking status. Binge drinking could not be included as this GWAS was also conducted in the UK Biobank.

##### Sensitivity analyses

The Cochran’s *Q* test of heterogeneity was conducted to estimate possible pleiotropy and the MR Egger intercept was estimated to test for bias from directional horizontal pleiotropy. The regression dilution *I*^2^_GX_ was calculated to assess the suitability of the MR Egger method and a simulation and extrapolation (SIMEX) correction applied where necessary [55]. Steiger filtering was conducted to check for evidence of reverse causation [56]. The mean *F* statistic was calculated as an indicator of instrument strength, where *F* < 10 is considered to indicate a weak instrument. Where there was evidence for a causal effect, we conducted multivariable MR analysis [57] to explore possible pleiotropy via education and ADHD liability (see Additional file 1: Note S7 [58, 59]).

## Results

Levels of the exposures and outcomes were highly consistent between the full MoBa sample and the genotyped sub-sample (Table 1). Prevalence of smoking, high alcohol consumption, high caffeine consumption and BMI were greater in males than females prior to pregnancy and males were older on average than females. Fertility and reproductive outcomes did not differ between females and males, except for age at first birth which was older in males. Associations between the outcomes found that an older age at first birth was associated with having fewer children in total, longer time to conception, being more likely to miscarry and more likely to have infertility treatment. Having more children was associated with a shorter time to conception, being more likely to have experienced miscarriage and less likely to have used infertility treatment. A full table of associations between the outcomes is presented in Additional file 1: Table S2. In the following sections, we highlight results that passed Bonferroni correction. Results comparing planning and non-planning couples are given in Additional file 1: Note S1, Table S3-S5, results for dichotomised time to conception are given in Additional file 1: Note S2, and results for frequency of sexual intercourse are given in Additional file 1: Note S3, Table S6.

**Table 1:** Descriptive statistics comparing exposure and outcome data across females and males

	**Females**				**Males**			
	Full sample		Genotyped sample		Full sample		Genotyped sample	
	***N***	**Mean (SD)/%**	***N***	**Mean (SD)/%**	***N***	**Mean (SD)/%**	***N***	**Mean (SD)/%**
**Age** (years)	94,327	30.20 (4.69)	70,858	30.25 (4.64)	74,327	32.78 (5.40)	49,979	32.75 (5.28)
**BMI** (kg/m^2^)	84,120	24.06 (4.31)	63,660	24.09 (4.30)	68,126^a^	25.90 (3.35)	45,636^a^	25.92 (3.32)
**Alcohol consumption**								
*Total*	80,763	-	61,752	-	64,639	-	43,267	-
*Never*	5923	7.33%	4362	7.06%	1475	2.28%	845	1.95%
*Less than once a month*	24,164	29.92%	18,379	29.76%	11,414	17.66%	7404	17.11%
*1*–*3 times a month*	28,824	35.69%	22,187	35.93%	23,569	36.46%	15,900	36.75%
*Once per week*	14,400	17.83%	11,085	17.95%	15,949	24.67%	10,876	25.14%
*2*–*3 times per week*	6550	8.11%	5037	8.16%	10,355	16.02%	7043	16.28%
*4*–*5 times per week*	738	0.91%	575	0.93%	1512	2.34%	1001	2.31%
*6*–*7 times per week*	164	0.20%	127	0.21%	365	0.56%	198	0.46%
**Binge drinking**								
*Total*	79,863	-	61,136	-	31,258	-	21,145	-
*Never*	27,022	33.84%	20,483	33.50%	3357	10.74%	2130	10.07%
*Less than once a month*	31,478	39.42%	24,188	39.56%	13,175	42.15%	9039	42.75%
*1*–*3 times a month*	16,417	20.56%	12,723	20.81%	10,127	32.40%	6842	32.36%
*Once a week*	4318	5.41%	3292	5.38%	3875	12.40%	2661	12.58%
*Several times per week*	628	0.79%	450	0.74%	725	2.32%	473	2.24%
**Smoking initiation**								
*Total*	83,962	-	63,533	-	66,288	-	44,203	-
*Never smokers*	41,878	49.88%	31,439	49.48%	31,396	47.36%	21,276	48.13%
*Ever smokers*	42,084	50.12%	32,094	50.52%	34,892	52.64%	22,927	51.87%
**Smoking heaviness** (cigarettes per day)	16,431	11.37 (5.93)	12,617	11.38 (5.90)	12,727	13.42 (6.37)	8273	13.39 (6.18)
**Caffeine consumption (mg per day)**	76,310	141.17 (138.33)	57,913	141.57 (138.45)	29,998	125.32 (85.22)	20,254	125.52 (84.50)
**Age at first birth** (years)	94,643	27.15 (4.65)	71,005	27.19 (4.61)	74,599	29.61 (4.99)	50,084	29.65 (4.91)
**Number of children** (*N* children)	94,643	2.55 (0.95)	71,005	2.54 (0.93)	74,599	2.51 (0.91)	50,084	2.50 (0.88)
**Time to conception** (months)	65,828	4.86 (7.87)	50,344	4.88 (7.93)	54,889^b^	4.82 (7.77)	37,298^b^	4.81 (7.70)
**Infertility treatment**								
*Total*	84,850	-	64,236	-	-	-	-	-
*Never*	77,098	90.86%	58,302	90.76%	-	-	-	-
*Ever*	7752	9.14%	5934	9.24%	-	-	-	-
**Miscarriage**								
*Total*	58,206	-	44,267	-	-	-	-	-
*Never*	41,214	70.81%	31,333	70.78%	-	-	-	-
*Ever*	16,992	29.19%	12,934	29.22%	-	-	-	-

^a^Supplemented with mother’s report when father’s report was unavailable

^b^Father variable reported by the mother

### Stage 1. Multivariable regression associations (MoBa)

**Fig. 1:**
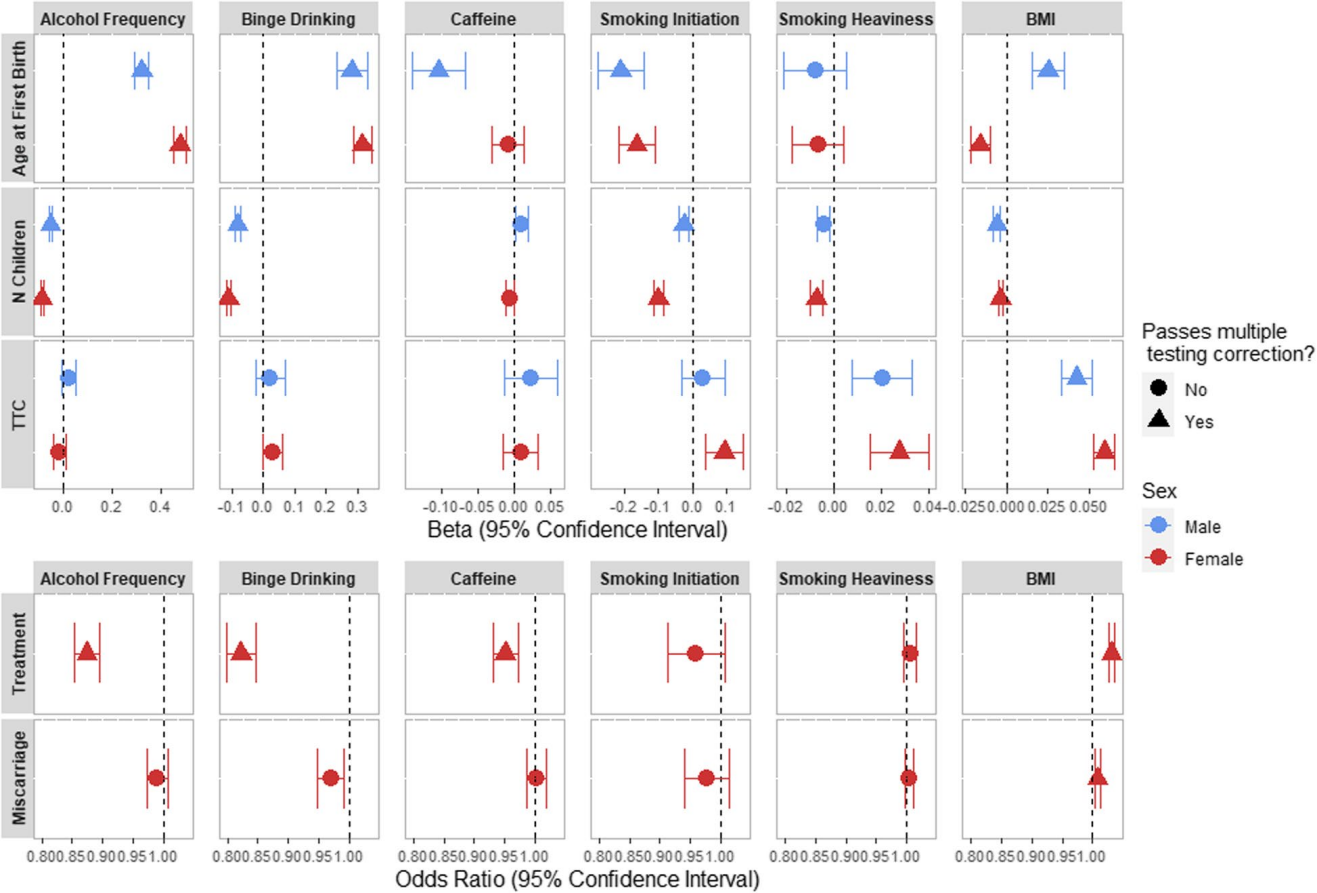
The association between health behaviours with fertility and reproductive outcomes from multivariable regression analyses comparing females and males. For continuous outcomes, units are betas, interpreted as mean difference in the outcome per unit increase in exposure (or the difference between ever smokers and non-smokers for smoking initiation). For binary outcomes, multivariable regression units are odds ratios. BMI = Body Mass Index, Caffeine = Caffeine Consumption, N Children = Number of Children, TTC = Time to Conception, Treatment = Use of Infertility Treatment

The results of the observed associations between the health behaviours and the fertility and reproductive outcomes (adjusted for birth year and education) are given in Fig. 1 (Additional file 1: Tables S7-S8).

#### Alcohol consumption

Units are per category increase in self-reported alcohol consumption or binge drinking. For reproductive outcomes, greater frequency of alcohol consumption and binge drinking were both associated with having fewer children (female alcohol frequency: − 0.091 children, 95% CI: − 0.096, − 0.085; female binge drinking: − 0.111 children, 95% CI: − 0.118, − 0.104; male alcohol frequency: − 0.055 children, 95% CI: − 0.062, − 0.049; male binge drinking: − 0.081 children, 95% CI: − 0.092, − 0.071) and an older age at first birth (female alcohol frequency: 0.479 years, 95% CI: 0.455, 0.503; female binge drinking: 0.317 years, 95% CI: 0.289, 0.346; male alcohol frequency: 0.322 years, 95% CI: 0.292, 0.352; male binge drinking: 0.283 years, 95% CI: 0.235, 0.331). For fertility outcomes, those who consumed more alcohol were less likely to have had infertility treatment (alcohol frequency: OR = 0.875, 95% CI: 0.854, 0.895; binge drinking: OR = 0.822, 95% CI: 0.798, 0.847). Neither alcohol frequency nor binge drinking were associated with miscarriage or time to conception after correction for multiple testing.

#### Caffeine consumption

Units are per unit increase in log transformed mg of caffeine per day. Higher caffeine consumption was associated with a younger age at first birth in males (− 0.103 years, 95% CI: − 0.140, − 0.066) and being less likely to have infertility treatment in females (OR = 0.951, 95% CI: 0.931, 0.972). Caffeine consumption was not associated with any of the other outcomes in males nor in females after correction for multiple testing.

#### Smoking behaviour

For reproductive outcomes, ever smoking was associated with having fewer children (females: − 0.098 children, 95% CI: − 0.111, − 0.086; males: − 0.024 children, 95% CI: − 0.038, − 0.010) and a younger age at first birth (females: − 0.161 years, 95% CI: − 0.213, − 0.110; males: − 0.209 years, 95% CI: − 0.275, − 0.143). Smoking heaviness (cigarettes per day) was only associated with having fewer children in females after correction for multiple testing (− 0.008 children, 95% CI: − 0.010, − 0.005) For fertility outcomes, both smoking measures were associated with an increased time to conception in females (smoking initiation: 0.094 months, 95% CI: 0.039, 0.149; smoking heaviness: 0.028 months, 95% CI: 0.015, 0.040).

#### Body mass index

Units are per kg/m^2^ increase in BMI. For reproductive outcomes, higher BMI was associated with having fewer children (females: − 0.004 children, 95% CI: − 0.005, − 0.002; males: − 0.006 children, 95% CI: − 0.008, − 0.004). In males, higher BMI was associated with an older age at first birth (0.025 years, 95% CI: 0.016, 0.035) but with a younger age of first birth in females (− 0.016 years, 95% CI: − 0.022, − 0.010). For fertility outcomes, higher BMI was associated with taking longer to conceive (females: 0.059 months, 95% CI: 0.053, 0.066; males: 0.043 months, 95% CI: 0.033, 0.052), being more likely to have infertility treatment (OR: 1.032, 95% CI: 1.026, 1.037) and being more likely to have a miscarriage (OR: 1.008, 95% CI: 1.004, 1.013).

These results were relatively consistent compared to those observed when restricting the analysis to the genotyped sample only (Additional file 1: Tables S9 and S10) and after adjustment for ADHD traits (Additional file 1: Tables S7-S10).

### Stage 2. Individual-level mendelian randomisation (MoBa)

**Fig. 2:**
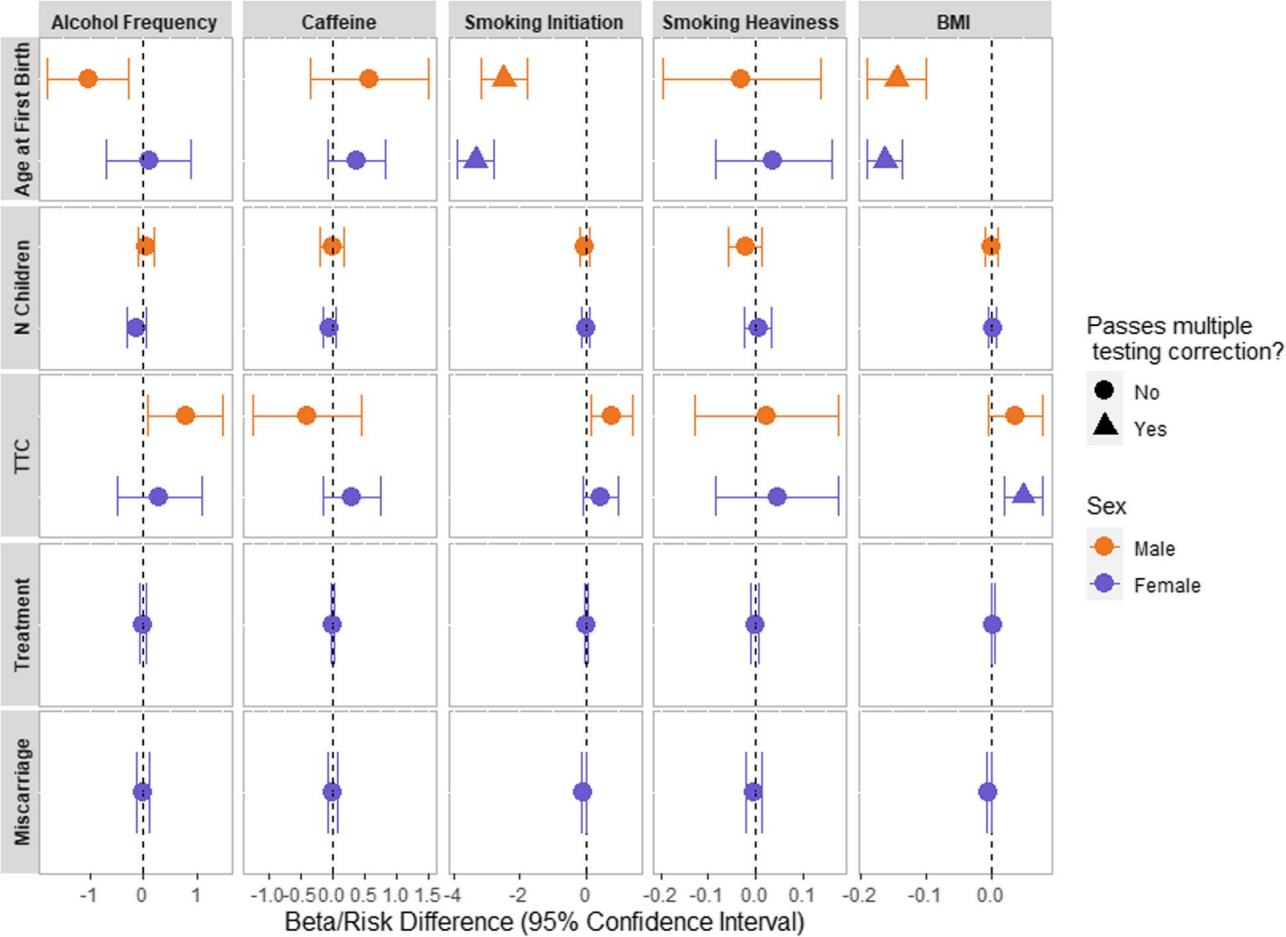
The association between health behaviours with fertility and reproductive outcomes from individual-level Mendelian randomisation analyses comparing females and males. Units of association can be interpreted as follows: for continuous exposures on continuous outcomes, units are mean difference in the outcome per unit increase in the genetically predicted exposure. For continuous exposures on binary outcomes, units approximate the risk difference of the outcome per unit increase in the genetically predicted exposure. The only exception is when smoking initiation is the exposure, which is binary. Therefore, exposure units are per standard deviation increase in the probability of being a smoker. Some 95% confidence intervals are narrow and therefore are not easily visible on the plot. BMI = Body Mass Index, Caffeine = Caffeine Consumption, N Children = Number of Children, TTC = Time to Conception, Treatment = Use of Infertility Treatment

Genetic scores were associated with the exposures in MoBa with the exception of binge drinking in males (Additional file 1: Table S1). Due to this and low power to detect effects in females (Additional file 1: Note S5), binge drinking was not included in the MR analyses. Results of the individual-level MR are presented in Fig. 2 (Additional file 1: Tables S11 for females and S12 for males).

For reproductive outcomes, genetically predicted higher BMI was associated with a younger age of first birth (females: − 0.162 years, 95% CI: − 0.189, − 0.136; males: − 0.145 years, 95% CI: − 0.189, − 0.100), as was genetic liability for smoking initiation (females: − 3.344 years, 95% CI: − 3.890, − 2.797; males: − 2.478 years, 95% CI: − 3.164, − 1.792). There was weak evidence for an association between genetically predicted higher alcohol consumption and younger age at first birth in males (− 1.024 years, 95% CI: − 1.785, − 0.263), but this did not survive correction for multiple testing.

For fertility outcomes, genetically predicted higher BMI was associated with increased time to conception in females (0.049 months, 95% CI: 0.020, 0.077). There was weak evidence for genetic liability to smoking initiation and genetically predicted higher alcohol consumption both associating with increased time to conception in males, but neither of these associations survived correction for multiple testing (smoking: 0.818 months, 95% CI: 0.186, 1.451; alcohol: 0.802 months, 95% CI: 0.097, 1.507). We found no robust evidence for association between any of the other exposures and outcomes using individual-level MR.

For BMI, smoking initiation and alcohol consumption genetic scores there was evidence of assortative mating (Additional file 1: Table S13) and associations between these genetic scores and other health behaviours and household income (Additional file 1: Table S14). Additional sensitivity analyses which are more robust to pleiotropy were consistent in direction of effect, with the exception of the weighted mode estimate for time to conception (Additional file 1: Table S15). This could suggest bias from pleiotropy, but the estimate was imprecise for this method due to low power and the MR Egger intercept showed no evidence of bias from directional horizontal pleiotropy (Additional file 1: Table S15).

There was no evidence for effects of smoking heaviness in the single-SNP analysis (Additional file 1: Note S4, Fig. S2. Results for individual-level MR restricting to couples first pregnancy can be found in Additional file 1: Note S6, Fig. S3-S4, Table S16-S17.

### Stage 3. Summary-level mendelian randomisation (UK Biobank)

There was strong evidence for an effect of smoking initiation on younger age at first birth in females (− 0.661, 95% CI: − 0.757, − 0.566), a greater number of children in both males and females (0.280, 95% CI: 0.205, 0.355) and fewer miscarriages in females (− 0.123, 95% CI: − 0.182, − 0.064) (see Table 2). All pleiotropy robust sensitivity analyses showed consistently strong evidence with the same direction of effect. There was evidence of significant heterogeneity (Additional file 1: Table S18), but the MR Egger intercept suggested that these results were not biased by directional pleiotropy (Additional file 1: Table S19). Steiger filtering indicated that the majority of SNPs explained more variance in smoking initiation than the outcomes (Additional file 1: Table S20), suggesting reverse causation is unlikely.

**Table 2:** Summary level Mendelian randomisation results for health behaviours on age at first birth, number of children and number of miscarriages

**Exposure**	**Outcome**	**Method**	***N* SNP**	**Beta (95% CI)**	***P***
**Drinks per week**	Age at first birth	IVW	94	− 0.055 (− 0.145, 0.034)	0.22
		MR Egger	94	0.04 (− 0.095, 0.175)	0.56
		Weighted median	94	− 0.048 (− 0.139, 0.042)	0.30
		Weighted mode	94	− 0.046 (− 0.129, 0.038)	0.29
	Number of children	IVW	83	0.027 (− 0.057, 0.110)	0.53
		MR Egger (SIMEX)	83	0.014 (− 0.029, 0.058)	0.52
		Weighted median	83	− 0.010 (− 0.094, 0.074)	0.82
		Weighted mode	83	0.009 (− 0.068, 0.086)	0.82
	Number of miscarriages	IVW	94	− 0.029 (− 0.097, 0.038)	0.39
		MR Egger	94	− 0.018 (− 0.121, 0.084)	0.73
		Weighted median	94	0.001 (− 0.099, 0.102)	0.98
		Weighted mode	94	0.01 (− 0.084, 0.103)	0.84
**Caffeine**	Age at first birth	IVW	6	− 0.036 (− 0.13, 0.057)	0.45
		Weighted median	6	0.013 (− 0.073, 0.099)	0.77
		Weighted mode	6	0.033 (− 0.067, 0.134)	0.54
	Number of children	IVW	6	0.021 (− 0.031, 0.073)	0.43
		Weighted median	6	0.021 (− 0.047, 0.089)	0.54
	Number of miscarriages	IVW	6	− 0.042 (− 0.106, 0.022)	0.20
		Weighted median	6	− 0.052 (− 0.128, 0.025)	0.19
		Weighted mode	6	− 0.076 (− 0.178, 0.025)	0.20
**Smoking initiation**	Age at First Birth	IVW	322	− 0.661 (− 0.757, − 0.566)	3.57 × 10^−42^
		Weighted median	322	− 0.504 (− 0.603, − 0.405)	2.71 × 10^−23^
		Weighted mode	322	− 0.555 (− 0.785, − 0.326)	3.30 × 10^−6^
	Number of children	IVW	323	0.280 (0.205, 0.355)	3.17 × 10^−13^
		Weighted median	323	0.207 (0.125, 0.289)	7.11 × 10^−07^
		Weighted mode	323	0.177 (− 0.009, 0.363)	0.06
	Number of miscarriages	IVW	322	− 0.123 (− 0.182, − 0.064)	4.68 × 10^−5^
		Weighted median	322	− 0.106 (− 0.196, − 0.017)	0.019
		Weighted mode	322	− 0.176 (− 0.399, 0.047)	0.124
**BMI**	Age at first birth	IVW	95	− 0.108 (− 0.16, − 0.056)	4.32 × 10^−05^
		MR Egger	95	− 0.076 (− 0.225, 0.072)	0.32
		Weighted median	95	− 0.043 (− 0.101, 0.015)	0.14
		Weighted mode	95	0.006 (− 0.114, 0.126)	0.92
	Number of children	IVW	91	− 0.014 (− 0.054, 0.027)	0.51
		MR Egger	91	− 0.002 (− 0.117, 0.113)	0.98
		Weighted median	91	− 0.008 (− 0.054, 0.038)	0.73
		Weighted mode	91	− 0.013 (− 0.094, 0.069)	0.76
	Number of miscarriages	IVW	95	0.004 (− 0.033, 0.041)	0.83
		MR Egger	95	0.021 (− 0.086, 0.128)	0.70
		Weighted median	95	0.019 (− 0.033, 0.072)	0.47
		Weighted mode	95	0.013 (− 0.091, 0.116)	0.81

*IVW*, inverse-variance weighted; *BMI*, body mass index. The I^2^_GX_ suggested that the smoking initiation and caffeine genetic instruments were not suitable for conducting MR Egger (Additional file 1: Table S21). Unweighted SIMEX corrections were conducted for smoking initiation. Age at first birth and number of miscarriages are in females only. Number of children is in males and females combined

There was also some evidence for an effect of higher BMI on younger age at first birth in females (− 0.108, 95% CI: − 0.16, − 0.056). All MR sensitivity analyses showed a consistent direction of effect apart from the weighted mode which could indicate possible pleiotropy (Table 2). There was strong evidence of heterogeneity (Additional file 1: Table S18), but the MR Egger intercept did not suggest this was due to bias from horizontal pleiotropy (Additional file 1: Table S19), and there was no evidence of reverse causation from Steiger filtering (Additional file 1: Table S20). There was no robust evidence for an effect of alcohol consumption or caffeine consumption on reproductive outcomes. All genetic instruments had *F*-statistic > 10 apart from that for smoking initiation (Additional file 1: Table S21).

We conducted multivariable MR analysis [57] to estimate the direct effects of smoking initiation and BMI on age at first birth in females after accounting for education and ADHD liability (Additional file 1: Table S22). Effect estimates for smoking initiation attenuated after adjustment but there was still evidence for an effect (after adjusting for ADHD: − 0.435, 95% CI: − 0.591, − 0.279; after adjusting for education: − 0.403, 95% CI: − 0.527, − 0.279). Effect estimates for BMI were also attenuated, resulting in weak evidence for an effect (ADHD: − 0.513, 95% CI: − 0.106, 0.003; education: − 0.056, 95% CI: − 0.113, 0.0008).

## Discussion

This study explored the role of multiple health behaviours on reproductive and fertility outcomes in pregnant females and their partners from the MoBa cohort, with replication in the UK Biobank. We extended previous research by including males as well as females and triangulating observational associations with Mendelian randomisation. We first discuss the associations with fertility outcomes and follow with the reproductive outcomes.

### Fertility outcomes

Multivariable regressions provided strong evidence for associations between higher BMI and worsened fertility outcomes: taking longer to conceive, increased likelihood of infertility treatment and increased risk of miscarriage. Previous evidence suggests that these associations are due to hormone imbalances and ovulatory dysfunction [60]. In female individual-level MR analysis, we found evidence for a causal effect of genetically predicted higher BMI on increased time to conception, supporting the observational association. This effect remained using the dichotomised measure of subfertility. However, there was no robust evidence in MR analysis for an association with the other fertility outcomes, nor for time to conception in males. This could be due to confounding, selection bias, low power to detect small effects or due to the two methods identifying different parameters. The multivariable regression estimates the average treatment effect, while MR estimates the local average treatment effect (an effect in those whose exposure would differ if the value of the instrumental variable differed [41]). Furthermore, MR analysis is unable to detect non-linear effects, which have previously been reported for BMI and subfertility in the MoBa cohort [61]. Evidence is also mixed for intervention studies, with a meta-analysis including randomised control trials finding evidence that interventions for weight loss increased chance of pregnancy but did not affect risk of miscarriage [62].

We found evidence in females for an association between smoking behaviours (smoking heaviness and smoking initiation) with increased time to conception in multivariable regression analyses. This is supported by a previous meta-analysis finding increased odds of infertility in smokers compared to non-smokers [6], with smoking hypothesised to affect the follicles and hormone levels in females [63]. In males, smoking is hypothesised to negatively affect sperm production and quality [64]. In the current study, we found weak evidence for an association between smoking heaviness and increased time to conception (but it did not survive correction for multiple testing). Furthermore, in the individual-level MR analysis, we found weak evidence for an association between smoking initiation and increased time to conception in males, although again this did not pass correction for multiple testing. A review of studies into health behaviours and fertility concluded that the evidence was most robust for smoking and higher weight reducing fertility [63]. This pattern is reflected in the current study, with these two traits being associated with time to conception across different study designs (although not consistently across all sexes and measures). It is also important to note that smoking and BMI were the two traits for which we had power to detect small effects, and even with the greatest power, smoking associations were weak, suggesting that effects are likely to be small.

Previous meta-analyses have shown a dose–response relationship between alcohol consumption and reduced likelihood of conception in females [5] and reduced semen quality in males [14]. In contrast, our study found that frequency of alcohol consumption was instead associated with being less likely to have infertility treatment in multivariable regression analyses for both males and females. However, levels of alcohol consumption were low in our sample, and are possibly below the threshold which affects fertility [63]. To capture more harmful drinking behaviours, we used a measure of binge drinking, which showed weaker associations, supporting our interpretation. It might be feasible that low levels of alcohol consumption are not detrimental for fertility, but it seems unlikely that alcohol could improve fertility, as observed here. Highly confounded multivariable regression associations of alcohol consumption are a common phenomenon, with low levels of consumption often being associated with positive outcomes due to confounding by socio-economic position or due to never drinkers being a selected group [65]. Bias from confounding is supported by our MR analyses, finding weak evidence for an effect of higher alcohol consumption on increased time to conception in males. This effect did not survive correction for multiple testing; however, the opposing direction of effect to multivariable regression analyses supports highly socioeconomically confounded observations of alcohol consumption. There was no robust evidence for associations between alcohol consumption and any of the other fertility traits using individual-level MR, although it is important to note that the genetic instrument for alcohol consumption only had reasonable power to detect moderate effect sizes, and hence small effects could have been missed.

There was no robust evidence for an association between caffeine consumption and miscarriage risk, in contrast to what has been reported in several meta-analyses [66]. This could be because our study explored reported caffeine consumption levels prior to pregnancy rather than during pregnancy, which has been the primary focus of most previous meta-analyses [66]. Alternatively, it could be due to social patterning of caffeine consumption in Norway, with higher consumption associated with lower levels of education [24] and consequently a younger age at first birth. Older age is a strong predictor of miscarriage risk [67], so education could be masking the association. MR results (which are more robust to bias from confounding) did not find evidence for a causal effect, so it could be possible that previous associations were due to confounding from other lifestyle factors [68]. However, it is important to note that the caffeine genetic instrument had the lowest power, with the genetic instrument explaining only 0.2–0.3% of the variance in the MoBa sample. Due to this weak instrument bias, small causal effects cannot be ruled out [69] and replication is warranted.

### Reproductive outcomes

While related to fertility, the reproductive outcomes of age at first birth and number of children are also highly socio-economically and behaviourally influenced. In multivariable regression analyses, higher levels of all health behaviours (except caffeine consumption) were associated with having fewer children. In individual-level MR analysis, there was no association between any of the health behaviours and number of children. This was the same for summary-level MR in UK Biobank, suggesting that observational associations could be due to socio-economic confounding. The only exception was strong evidence for an effect of genetic liability to smoking initiation on having more children in summary-level MR. This direction of effect is in contrast to the weak evidence for an effect on longer time to conception, highlighting that these reproductive outcomes are likely influenced by additional factors and could act via different (perhaps opposing) mechanisms.

In both the individual-level and summary-level MR, we saw consistent evidence for an effect of smoking initiation and higher BMI on having a younger age at first birth. It is possible that these results are due to selection bias: reporting age at first birth is conditioned on having had at least one child. If BMI and smoking are associated with reduced fertility, perhaps only those who had children younger were able to conceive and consequently be in the sample. This is supported by evidence for effects of BMI and weak evidence for effects of smoking on longer time to conception. An alternative explanation could be that our estimates are biased by horizontal pleiotropy, which we explored with a range of sensitivity analyses. Methods which are agnostic to the specific sources of pleiotropy (for example, MR-Egger) suggested that the results were not importantly biased by unbalanced horizontal pleiotropy. However, our exploratory multivariable MR analyses did show strong attenuation of the effects of BMI and smoking initiation on age at first birth after accounting for ADHD liability and educational attainment (known predictors of age at first birth). This is likely because ADHD traits affect smoking behaviour and some of the smoking genetic variants relate to smoking via their relationship to ADHD traits. Previous studies have shown a strong association between the smoking initiation instrument and risk-taking behaviours including number of sexual partners [70], which could increase the likelihood of having children younger. Previous Mendelian randomisation studies have also found evidence for bi-directional causal effects between smoking and education [71, 72], smoking and ADHD [73], BMI and education [74, 75] and BMI and ADHD [76]. Bi-directional effects between the exposure and the non-exposure traits can make it difficult to disentangle horizontal from vertical pleiotropy [77]. However, several of these previous studies did find evidence of horizontal pleiotropy, especially for the smoking initiation instrument [72, 73, 76]. Therefore, we conclude that horizontal pleiotropy is the most plausible. If there is indeed horizontal pleiotropy from ADHD liability and education, then direct effects from MVMR accounting for these traits will be closer to the true causal effect.

### Strengths and limitations

The current study has several strengths. The majority of epidemiological research to date has focused on females [13], but we also included males in our analysis. Second, we combined multivariable regression and MR methods which each rely on different assumptions and therefore triangulating across them can strengthen causal inference. Finally, we used a large sample of genotyped individuals with detailed measures of a range of different health behaviours, fertility outcomes and reproductive outcomes, influenced by a range of biological, social and behavioural factors.

This study does have several limitations. First, all MoBa participants were recruited during pregnancy (12–18 weeks gestation). This means that we are unable to capture the full range of fertility in the population. Those who never managed to conceive were not observed, and this could induce selection bias. We should be cautious to generalise the results beyond those who have been able to conceive. However, the outcomes of age at first birth, number of children and miscarriage risk were available for replication in the UK Biobank using summary-level MR. This sample is not selected on pregnancy and results were relatively consistent, suggesting that there is not substantial bias from selection on conception. Second, it is important to note that only smoking initiation and BMI exposures were powered to detect small effects in individual-level MR analyses. For fertility outcomes, small effects could still be meaningful and therefore, the absence of an association in the individual-level MR analysis should not be interpreted as evidence of absence of an effect. When even larger sample sizes and stronger genetic instruments are available, then analyses should be replicated. Third, multivariable regression analyses were cross-sectional, and it is therefore difficult to assess temporality for these associations. Specifically, health behaviours were retrospectively reported about behaviours 3 or 6 months prior to the index pregnancy; however, some couples had been trying to conceive for longer than 6 months. Furthermore, variables from the Medical Birth Registry of Norway (age at first birth and number of children) are across all births, and therefore, health behaviours may have differed compared to before the index pregnancy. Relatedly, there was also a difference between those in the sample who were planning to conceive compared with those who were not. We hypothesise that planners are more likely to be cautious about their health behaviours, especially if they have been having trouble conceiving and have been advised to quit smoking, stop drinking and lose weight. This could lead to reverse or weakened patterns of association in the multivariable regression analyses. However, MR would be robust to this type of bias, given that genetic propensities to health behaviours are fixed at birth. This might explain the different pattern of results between the multivariable regression and MR analyses. Fourth, for this paper, we have assumed that partners in MoBa were male. In our genetic analyses, this is the case, because individuals who were not chromosomally XY were removed from analysis. However, in the observational analysis, a small number of partners might have been female partners of the mother, and these individuals could not be identified. Finally, there was also strong evidence for assortative mating for both BMI and smoking initiation instruments which can bias MR results, even for methods which are robust to horizontal pleiotropy [78].

## Conclusions

For accurate fertility guidance, it is extremely important to establish causality. Our results can contribute to the evidence base upon which these decisions are made. Associations between higher BMI and smoking on increased time to conception were replicated using MR (although weaker for smoking), aligning with previous conclusions that the evidence is most robust for these traits. However, smoking and BMI were the best powered exposures in the MoBa sample, and small (but meaningful) effects of the other health behaviours cannot be ruled out. Replication is warranted when larger sample sizes (unselected on pregnancy) and more powerful instruments are available. There was evidence for a possible causal effect of smoking initiation and BMI on age at first birth (in a contradictory direction to results for time to conception). We found evidence of potential horizontal pleiotropy, as our genetic instruments for smoking initiation and BMI were also capturing educational attainment and ADHD liability. Therefore, triangulation across a broader range of methods, including those not susceptible to pleiotropy, is required to establish causality.

## Supplementary Information

**Additional file 1: ** **Fig. S1.** An overview of participant inclusion and attrition. **Fig. S2.** The influence of rs16969968 genotype on reproductive outcomes. **Fig. S3.** MR analyses comparing first pregnancy with full sample in females. **Fig. S4.** MR analyses comparing first pregnancy with full sample in males. **Note S1.** Comparison between planning and non-planning couples. **Note S2.** Sensitivity analysis dichotomising time to conception. **Note S3.** Health behaviours and frequency of sexual intercourse. **Note S4.** Single-SNP analysis of rs16969968 genotype stratified by smoking status. **Note S5.** Power calculation for Mendelian randomisation. **Note S6.** MR analyses restricting to couples with first pregnancy. **Note S7.** Genetic instruments for educational attainment and ADHD. **Table S1.** Testing instrument strength of each of the polygenic risk scores. **Table S2.** Associations between reproductive outcomes. **Table S3.** Summary of characteristics comparing planners and non-planners. **Table S4.** Including non-planners in time to conception observational analysis. **Table S5.** Including non-planners in time to conception MR. **Table S6.** Frequency of self-reported sexual intercourse. **Table S7.** Observational associations between health behaviours in females. **Table S8.** Observational associations between health behaviours in males. **Table S9.** Observational associations between health behaviours in genotyped females. **Table S10.** Observational associations between health behaviours in genotypes males. **Table S11.** Individual-level Mendelian randomisation analysis in the females. **Table S12.** Individual-level Mendelian randomisation analysis in the males. **Table S13.** Evidence for assortative mating. **Table S14.** Evidence for reintroduced confounding. **Table S15.** Summary level MR sensitivity tests conducted in the MoBa sample. **Table S16.** Comparison of MR results in females between first pregnancy and full sample. **Table S17.** Comparison of MR results in males between first pregnancy and full sample. **Table S18.** Evidence for heterogeneity: Cochran’s Q statistics. **Table S19.** The MR Egger intercept test: Evidence for bias from horizontal pleiotropy. **Table S20.** Steiger filtering test for possible reverse causation. **Table S21.** Test of instrument strength and the suitability of the instrument for MR Egger. **Table S22.** Exploratory multivariable Mendelian randomisation.

## Data Availability

The consent and ethical approvals for MoBa does not allow storage of data in repositories or journals, but it is possible to apply for access to summary statistics datasets for replication or reproduction of studies by sending an application to datatilgang@fhi.no. Data access requires approval from The Regional Committee for Medical and Health Research Ethics in Norway and an agreement with MoBa. All GWAS summary statistics are publicly available.

## Abbreviations

ADHD: Attention deficit hyperactivity disorder
BMI: Body mass index
GWAS: Genome-wide association study
MBRN: Medical Birth Registry of Norway
MoBa: Norwegian Mother, Father and Child Cohort Study
MR: Mendelian randomisation
QC: Quality control
SD: Standard deviation
SIMEX: Simulation and extrapolation
SNP: Single nucleotide polymorphism
