# Supplementary material for: Associations between health behaviours, fertility and reproductive outcomes: triangulation of evidence in the Norwegian Mother, Father and Child Cohort Study (MoBa)
Source: BMC Med. 2023 Apr 3;21:125. doi: 10.1186/s12916-023-02831-9 (PMC10071662; doi:10.1186/s12916-023-02831-9)
Supplement: Supplementary file 1 — Additional file 1: Fig. S1. An overview of participant inclusion and attrition. Fig. S2. The influence of rs16969968 genotype on reproductive outcomes. Fig. S3. MR analyses comparing first pregnancy with full sample in females. Fig. S4. MR analyses comparing first pregnancy with full sample in males. Note S1. Comparison between planning and non-planning couples. Note S2. Sensitivity analysis dichotomising time to conception. Note S3. Health behaviours and frequency of sexual intercourse. Note S4. Single-SNP analysis of rs16969968 genotype stratified by smoking status. Note S5. Power calculation for Mendelian randomisation. Note S6. MR analyses restricting to couples with first pregnancy. Note S7. Genetic instruments for educational attainment and ADHD. Table S1. Testing instrument strength of each of the polygenic risk scores. Table S2. Associations between reproductive outcomes. Table S3. Summary of characteristics comparing planners and non-planners. Table S4. Including non-planners in time to conception observational analysis. Table S5. Including non-planners in time to conception MR. Table S6. Frequency of self-reported sexual intercourse. Table S7. Observational associations between health behaviours in females. Table S8. Observational associations between health behaviours in males. Table S9. Observational associations between health behaviours in genotyped females. Table S10. Observational associations between health behaviours in genotypes males. Table S11. Individual-level Mendelian randomisation analysis in the females. Table S12. Individual-level Mendelian randomisation analysis in the males. Table S13. Evidence for assortative mating. Table S14. Evidence for reintroduced confounding. Table S15. Summary level MR sensitivity tests conducted in the MoBa sample. Table S16. Comparison of MR results in females between first pregnancy and full sample. Table S17. Comparison of MR results in males between first pregnancy and full sample. Table S18. Evidence for h [file 12916_2023_2831_MOESM1_ESM.docx]

**Additional File 1: Supplementary Materials**

**Contents.**

| Supplementary Figures  Fig. S1. An overview of participant inclusion and attrition.  Fig. S2. The influence of rs16969968 genotype on reproductive outcomes  Fig. S3. MR analyses comparing first pregnancy with full sample in females  Fig. S4. MR analyses comparing first pregnancy with full sample in males  Supplementary Notes | Page 3  Page 4  Page 5  Page 6 |
| --- | --- |
| Note S1. Comparison between planning and non-planning couples | Page 7 |
| Note S2. Sensitivity analysis dichotomising time to conception | Page 9 |
| Note S3. Health behaviours and frequency of sexual intercourse | Page 11 |
| Note S4. Single-SNP analysis of rs16969968 genotype stratified by smoking status | Page 12 |
| Note S5. Power calculation for Mendelian randomisation  Note S6. MR analyses restricting to couples with first pregnancy  Note S7. Genetic instruments for educational attainment and ADHD | Page 13  Page 14  Page 15 |
| Supplementary Tables |  |
| Table. S1. Testing instrument strength of each of the polygenic risk scores | Page 16 |
| Table. S2. Associations between reproductive outcomes  Table. S3. Summary of characteristics comparing planners and non-planners  Table. S4. Including non-planners in time to conception observational analysis  Table. S5. Including non-planners in time to conception MR  Table. S6. Frequency of self-reported sexual intercourse  Table. S7. Observational associations between health behaviours in females | Page 17  Page 18  Page 19  Page 20  Page 21  Page 22 |
| Table. S8. Observational associations between health behaviours in males | Page 24 |
| Table. S9. Observational associations between health behaviours in genotyped females | Page 26 |
| Table. S10. Observational associations between health behaviours in genotypes males | Page 28 |
| Table. S11. Individual-level Mendelian randomisation analysis in the females | Page 30 |
| Table. S12. Individual-level Mendelian randomisation analysis in the males | Page 31 |
| Table. S13. Evidence for assortative mating | Page 32 |
| Table. S14. Evidence for reintroduced confounding | Page 33 |
| Table. S15. Summary level MR sensitivity tests conducted in the MoBa sample  Table. S16. Comparison of MR results in females between first pregnancy and full sample  Table. S17. Comparison of MR results in males between first pregnancy and full sample | Page 34  Page 35  Page 36 |
| Table. S18. Evidence for heterogeneity: Cochran’s Q statistics | Page 38 |
| Table. S19. The MR Egger intercept test: Evidence for bias from horizontal pleiotropy | Page 39 |
| Table. S20. Steiger filtering test for possible reverse causation | Page 40 |
| Table. S21. Test of instrument strength and the suitability of the instrument for MR Egger | Page 41 |
| Table. S22. Exploratory multivariable Mendelian randomisation | Page 42 |
|  |  |

**Figure S1. An overview of participant inclusion and attrition.**

**Note.** QC = quality control, MBRN = medical birth registry of Norway. Analysis samples represent the maximum number of individuals available for analysis. For specific N for each variable, see Table 1 in the main manuscript. Variables from the MBRN were parental age, parent’s age at first birth and total number of children. All other variables were from questionnaire data. For additional details of genotyping QC procedure, see (Corfield et al., 2022).

**Figure S2. The influence of rs16969968 genotype (A allele) on reproductive outcomes stratified by smoking status prior to pregnancy**

**
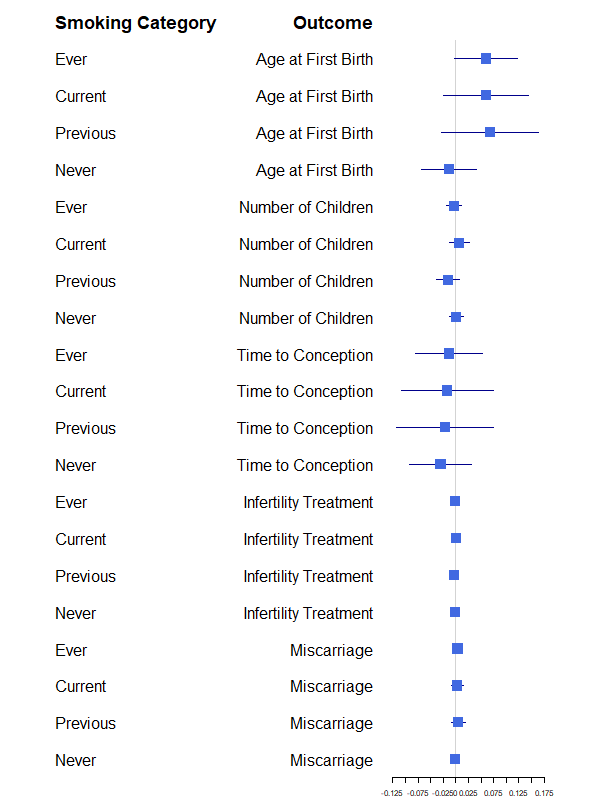
**

**Supplementary Figure S3. Individual-level MR analyses comparing first pregnancy with full sample in females**

**
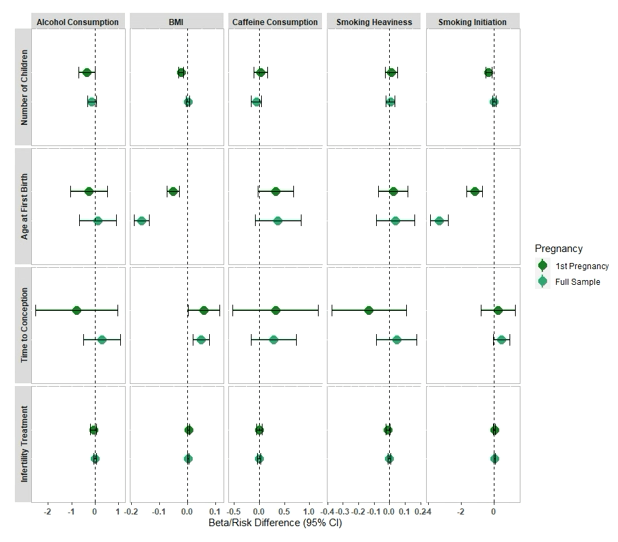
**

**Supplementary Figure S4. Individual-level MR analyses comparing first pregnancy with full sample in males**

**
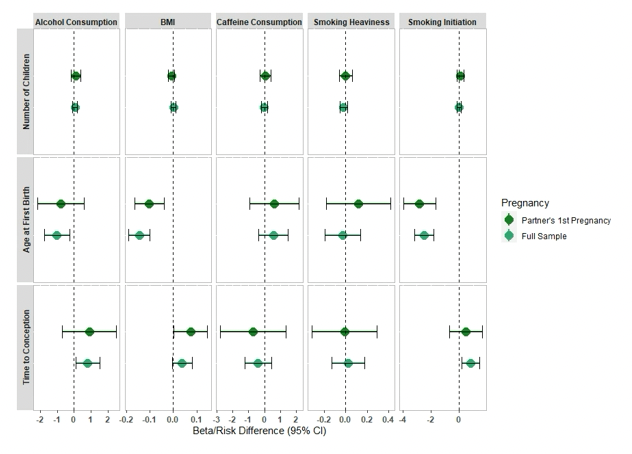
**

**Supplementary Note S1. Comparison between planners and non-planners**

Methods

Individuals who did not plan their pregnancy cannot provide a time to conception, as this will be unknown. In our primary analyses of time to conception, we have therefore excluded non-planners and included the planners only. Here we explore the difference between the couples who did and didn’t plan their pregnancy. First, we explore the demographic differences between the planning and the non-planning group. Second, we repeated our observational analysis and our individual-level MR analysis on time to conception but with the non-planners included. Non-planners cannot report time to conception, so we gave them the median time to conception from the planning group (2 months).

Results

Of the full sample, 15,600 (18.3%) reported that their pregnancy was unplanned and 69,788 (81.7%) reported that their pregnancy was planned. There are differences in demographics and health behaviours between the planning and the non-planning group (see TableS3). The non-planners are younger and less likely to be university educated. The non-planners had their first child younger, have more children, have lower BMI, smoke more, drink more alcohol and drink more caffeine. Therefore, bias will be introduced by including the planning group only.

In Table S4, we compare the primary observational results with an analysis where non-planners are included. There are disparities between the results. The associations between smoking initiation and smoking heaviness with increased time to conception were attenuated when including the non-planners. The association between higher alcohol consumption and reduced time to conception was strengthened when including the non-planners. The association between binge drinking and reduced time to conception was in fact reversed when including the non-planners, from increasing time to conception to decreasing it. Results remained relatively consistent for BMI and caffeine consumption.

In Table S5, we compare the individual-level MR results, with planners and non-planners included. When restricting to planners in the original analysis, only higher BMI was associated with increased time to conception. After the non-planners were included, the association was strengthened (mean difference in months per unit increase in genetically predicted BMI: 0.878, 95% CI: 0.776, 0.981). After the non-planners were included, there was evidence for genetically predicted higher alcohol consumption on a decreased time to conception (mean difference in months per unit increase in genetically predicted alcohol consumption: -1.991, 95% CI: -2.904, -1.078).

**Supplementary Note S2. Sensitivity analysis dichotomising time to conception**

Methods

In our primary analysis we used self-reported time to conception as an outcome variable. However, this measure was not linear, with response categories of less than one month, 1-2 months or 3+ months. If it took more than 3 months, then females were asked to state the number of months. We combined anyone taking 12 or more months to conceive into one group to reduce skewness and treated as a continuous variable. Given the skew in this variable, we performed a sensitivity analysis where we created two additional dichotomised variables: subfertility and high fertility.

*Subfertility****.*** Subfertility was defined as taking 12 months or longer to conceive. This was based upon females self-reported time to conception from the 15-week questionnaire. We have used this female-reported variable as an outcome in both females and males as couples were conceiving together.

*High fertility.* A second binary variable represented the other end of the fertility distribution. ‘Highly fertile’ couples were defined as conceiving in less than one month (if they were trying to conceive) or those who conceived despite using contraception (using any contraception methods apart from withdrawal or safe period unless in combination with another form of contraception). This was based upon females self-reported time to conception from the 15-week questionnaire. We have used this female-reported variable as an outcome in both females and males under the assumption that couples were trying to conceive together.

Results

*Descriptives.* In the full sample, 9.8% of females and 9.0% of males met criteria for subfertility, and 23.4% of females and 23.7% of males met criteria for high fertility. The proportions were the same in the genotyped sample to 1 decimal place.

*Observational associations.* Association estimates reported here are conducted in the full sample and adjusted for birthyear and education (Tables S7-S8). Higher BMI was associated with being more likely to have subfertility (females OR: 1.046, 95% CI: 1.040, 1.052; males OR: 1.034, 95% CI: 1.025, 1.043) and less likely to have high fertility (females OR: 0.987, 95% CI: 0.983, 0.991; males OR: 0.982, 95% CI: 0.976, 0.987). Higher smoking heaviness was associated with a higher likelihood of subfertility in both females (OR: 1.028, 95% CI: 1.017, 1.038) and males (OR: 1.020, 95% CI: 1.009, 1.031). Higher caffeine consumption, alcohol consumption and smoking initiation were not associated with either being highly fertile or odds of subfertility. Results were relatively consistent with those conducted in the genotyped sample only (Tables S9-S10).

*Individual-level MR.* We found no evidence for association between any of the health behaviours with either high fertility or subfertility after correcting for multiple testing, with the exception of higher BMI predicting an increased risk of subfertility in females (risk difference per unit increase in genetically predicted BMI: 0.006, 95% CI: 0.003, 0.008) (Tables S11-S12). Additional sensitivity analyses which are more robust to pleiotropy were consistent in direction of effect with the exception of the weighted mode method (Table S15). This could suggest bias from pleiotropy, but the estimate was imprecise for this method due to low power and the MR Egger intercept showed no evidence of bias from directional horizontal pleiotropy (Table S15).

**Supplementary Note S3. Exploring the association between health behaviours and frequency of sexual intercourse**

Methods

As a secondary outcome, we looked at the association between health behaviours and frequency of sexual intercourse. This is not a reproductive outcome per se but a reproductive behaviour which could be a possible mediator of effects on reproductive outcomes (e.g., time to conception, number of children etc.) and on fertility outcomes (e.g. time to conception).

*Variable definition.* Females self-reported (in the 15-week questionnaire) the frequency they had sexual intercourse in the 4 weeks prior to pregnancy. Response categories were on a 7-point scale from *never* to *every day*. Males were assigned their female partner’s self-reported frequency if they reported having been in a sexual relationship with their partner for 5 weeks or longer (to ensure they were together for 4 weeks prior to pregnancy). Otherwise, male frequency was set to missing.

Results

*Descriptives.* It was most common to have sex 1-2 times a week for both males (36.27%) and females (35.89%). Full response frequencies are given in Table S6.

*Observational associations.* In the full sample analyses (adjusted for birthyear and education), higher frequency of alcohol consumption and binge drinking were both associated with having sex more frequently in females (alcohol frequency: 0.072 (0.064, 0.079); binge drinking: 0.067 ( 0.058, 0.075)) and in males (alcohol frequency: 0.041 ( 0.033, 0.049); binge drinking: 0.077 ( 0.063, 0.091)). Higher caffeine consumption was associated with increased sex frequency in females (0.020 (0.013, 0.026)) but not males. Higher BMI in both males and females was associated with having sex less frequently (females: -0.007 (-0.008, -0.005) males: -0.008 (-0.010, -0.005)). Smoking heaviness was associated with having more frequent sexual intercourse in females (0.007 (0.004, 0.011)) and males (0.006 ( 0.002, 0.009)) (Tables S7-S8). Results were relatively consistent with those conducted in the genotyped sample only (Tables S9-S10).

*Individual-level Mendelian randomisation.* Genetic liability for any of the health behaviours was not associated with frequency of sexual intercourse (Tables S11 and S12).

**Supplementary Note S4. Single-SNP analysis of rs16969968 genotype stratified by smoking status**

Methods

We conducted an individual-level MR analysis using a single-nucleotide polymorphism (SNP) for smoking heaviness. We used the genetic variant rs16969968 (A/G) found in the gene cluster *CHRNA5-A3-B4*, a nicotinic receptor subunit gene cluster on chromosome 15 that has been robustly associated with smoking heaviness in smokers (Ducci et al., 2011; Munafò et al., 2012). This SNP is functional and leads to an amino acid change (D398N) in the nicotinic receptor α5 subunit protein (Fowler et al., 2011). The minor allele is associated with nicotine metabolism such that each allele increase in rs16969968 corresponds to an average of one more cigarette smoked per day (Munafò et al., 2012; Thorgeirsson et al., 2008; Tobacco Consortium, 2010). Therefore, if there were to be evidence for an effect in current smokers, this could suggest a causal role of smoking. An additional strength of this approach is being able to compare the effects in current smokers with the effects in never smokers. If there is any evidence for an effect in the never smokers, then this is indicative of horizontal pleiotropy, through pathways other than smoking (Millard et al., 2019).

Genotype at rs16969968 was coded as 0, 1 or 2, counting the number of minor alleles (A). We stratified our sample into ever smokers, previous smokers, current smokers and never smokers, as retrospectively reported (at 15-week questionnaire) about 3-months prior to pregnancy. A linear regression was run of rs16969968 genotype for each smoking status on each continuous reproductive outcome and a logistic regression was run for each binary outcome. Analysis was conducted in R, version 4.0.3.

Results

The results of the single SNP analysis are presented in Figure S2. There was no evidence for an effect of smoking heaviness on any of the reproductive outcomes, which would be indicated through an association in the current smokers and no association in the never smokers (the negative control). Results are presented as betas for continuous outcomes (per allele increase) and as risk differences for binary outcomes (per allele increase).

**Supplementary Note S5. Power Calculation for Individual-level Mendelian randomisation in the MoBa cohort**

We conducted power calculations using the mRnd power calculator (Brion et al., 2013) for individual-level Mendelian randomisation. We input the sample size available in the MoBa cohort for each comparison. For the variance explained by the instrument, we used the R^2^ (or pseudo R^2^ for smoking initiation) estimated within the MoBa cohort (Table S1). We kept the variance in the exposure and variance in the outcome constant at 1 and set the Type-I error rate to 0.05. In the table below, we present the smallest standardised beta coefficient that we have 80% power to detect.

| PRS | Sex | R^2^ | N | Beta 80% power |
| --- | --- | --- | --- | --- |
| BMI | Females | 0.067 | 62,545 | 0.04 |
|  | Males | 0.064 | 45,460 | 0.05 |
| Caffeine Consumption | Females | 0.003 | 56,874 | 0.21 |
|  | Males | 0.002 | 20,154 | 0.40 |
| Smoking Heaviness | Females | 0.010 | 12,140 | 0.25 |
|  | Males | 0.009 | 8,191 | 0.31 |
| Alcohol Consumption | Females | 0.0009 | 61,599 | 0.35 |
|  | Males | 0.002 | 43,092 | 0.29 |
| Binge drinking | Females | 0.0002 | 60,985 | 0.63 |
|  | Males | 0.0001 | 21,036 | 0.89 |
| Smoking Initiation | Females | 0.010 | 63,376 | 0.11 |
|  | Males | 0.009 | 44,025 | 0.14 |

If, similarly to a correlation coefficient, we interpret a standardised beta coefficient <0.2 to be small, <0.5 to be moderate and <0.5 to be large (Acock, 2014), then we have 80% to detect moderate effect sizes for all exposures other than binge drinking. Due to this very low power of binge drinking analyses in MoBa, we did not conduct individual-level MR analyses for this exposure. Furthermore, the binge drinking instrument does not significantly predict binge drinking behaviour in males (Table S1), and therefore does not satisfy the relevance assumption.

It is important to note that only for smoking initiation and BMI exposures are we powered to detect small effects. Small effects could definitely still be meaningful for our analyses and therefore, the absence of an association in the individual-level MR analysis should not be interpreted as support for the null hypothesis. When even larger sample sizes and stronger genetic instruments are available, then analyses should be replicated.

**Supplementary Note S6. Individual-level MR analyses restricting to couples with first pregnancy**

Methods

In the MoBa cohort, some parents already have children, and for others the Moba index pregnancy is their first child. Already having had another pregnancy or another child may influence the parent’s health behaviours, leading to bias in individual-level MR analyses from reverse causation by violation of the exclusion restriction assumption (Vanderweele et al., 2014). Therefore, we conducted a sensitivity analysis restricting to individuals for whom it was their first pregnancy. MoBa females were asked in the 15-week questionnaire “*Have you been pregnant before? (Include all pregnancies that ended in abortion, miscarriage or stillbirth)*”. We restricted to females (and their partners) who responded *no* to this item. We were not able to include miscarriage as an outcome for this sensitivity analysis because individuals were only asked this question if they had had a previous pregnancy.

Results

Of the total sample, 32% had not been pregnant before. This only left us with a sample of up to 19,507 females and 15,206 males. In females, there was consistency between estimates from the full sample and the sample restricted to first pregnancy, with the exceptions of BMI on age at first birth and smoking initiation on age at first birth. Both estimates were attenuated when restricting to only those for whom it was their first pregnancy (Figures S3, Tables S16). For males, the results were relatively consistent for all analyses between the full sample and the sample restricted to first pregnancy (Figure S4, Table S17).

**Supplementary Note S7. Genetic instruments for educational attainment and ADHD**

In multivariable MR analyses, the effects of smoking initiation and BMI were adjusted for educational attainment and liability to attention deficit hyperactivity disorder (ADHD). Details of the genome-wide association studies (GWAS) used to identify the relevant genetic instruments are detailed below.

Educational attainment

Educational attainment was measured as years in education. In a sample of 293,723 individuals, there were 74 independent genome-wide significant SNPs identified (Okbay et al., 2016).

Attention deficit hyperactivity disorder (ADHD)

A GWAS of attention deficit hyperactivity disorder (ADHD) diagnoses comprising 20,183 cases and 35,191 controls was used as a proxy for impulsivity (Demontis et al., 2019). There were 9 SNPs independently associated with ADHD at p<5x10-6.

Results are presented in Table S22.

**Supplementary Table S1. Testing instrument strength of the polygenic risk scores**

| PRS | Exposure | N SNP | R2/Pseudo R2 | Beta/OR (95% CI) | P-value |
| --- | --- | --- | --- | --- | --- |
| **Females** |  |  |  |  |  |
| Binge p<5x10-8 | Binge Drinking | 4 | 0.0002 | 0.011 (0.004, 0.019) | 0.002 |
| BMI p<5x10-8 | BMI | 995 | 0.067 | 1.112 (1.080, 1.145) | <0.001 |
| Caffeine p<5x10-8 | Caffeine Consumption | 6 | 0.003 | 0.067 (0.057, 0.077) | <0.001 |
| CPD p<5x10-8 | Smoking Heaviness | 39 | 0.010 | 0.596 (0.494, 0.698) | <0.001 |
| DPW p<5x10-8 | Alcohol Frequency | 66 | 0.0009 | 0.034 (0.025, 0.042) | <0.001 |
| SI p<5x10-8 | Smoking Initiation | 283 | 0.010 | 1.273 (1.253, 1.293) | <0.001 |
| **Males** |  |  |  |  |  |
| Binge p<5x10-8 | Binge Drinking | 4 | 0.0001 | 0.009 (-0.003, 0.021) | 0.152 |
| BMI p<5x10-8 | BMI | 995 | 0.064 | 0.841 ( 0.811, 0.870) | <0.001 |
| Caffeine p<5x10-8 | Caffeine Consumption | 6 | 0.002 | 0.051 ( 0.035, 0.068) | <0.001 |
| CPD p<5x10-8 | Smoking Heaviness | 39 | 0.009 | 0.589 ( 0.457, 0.721) | <0.001 |
| DPW p<5x10-8 | Alcohol Frequency | 66 | 0.002 | 0.049 ( 0.038, 0.059) | <0.001 |
| SI p<5x10-8 | Smoking Initiation | 283 | 0.009 | 1.258 ( 1.235, 1.283) | <0.001 |

Note. Variance explained is presented as R^2^ for continuous exposures and as pseudo R^2^ for the binary exposure (smoking initiation). SI = smoking initiation, DPW = drinks per week, CPD = cigarettes per day, BMI = body mass index, N SNP = the number of SNPs available in the sample to construct the score after clumping.

**Supplementary Table S2. Associations between fertility and reproductive outcomes in the full sample.**

| **Females** | AFB (years) | N Children | TTC (months) | Sex Freq | Miscarriage | Infertility Treatment |
| --- | --- | --- | --- | --- | --- | --- |
| Age at first birth |  | 94,643 | 65,748 | 84,368 | 58,175 | 84,788 |
| Number of children | r=-0.34  p<0.001 |  | 65,748 | 84368 | 58,175 | 84,788 |
| Time to Conception | r=0.11  p<0.001 | r=-0.08  p<0.001 |  | 65,001 | 45,370 | 65,476 |
| Frequency of sexual intercourse | r=-0.17  p<0.001 | r=0.05  p<0.001 | r=-0.08  p<0.001 |  | 57,269 | 83,481 |
| Miscarriage | Never = 26.61  Ever = 27.56  p<0.001 | Never = 2.67  Ever = 2.76  p<0.001 | Never = 4.12  Ever = 5.58  p<0.001 | Never = 4.29  Ever = 4.35  p<0.001 |  | 57,633 |
| Infertility treatment | Never = 27.05  Ever = 29.76  p<0.001 | Never = 2.56  Ever = 2.37  p<0.001 | Never = 3.79  Ever = 17.65  p<0.001 | Never = 4.41  Ever = 4.09  p<0.001 | $\chi^{2}$= 578 (df=1)  p<0.001 |  |
|  |  |  |  |  |  |  |
| **Males** | AFB | N Children | TTC | Sex Freq |  |  |
| Age at first birth |  | 74,599 | 54,815 | 67,698 |  |  |
| Number of children | r=-0.36  p<0.001 |  | 54,815 | 67,698 |  |  |
| Time to Conception | r=0.10  p<0.001 | r=-0.06  p<0.001 |  | 53,931 |  |  |
| Frequency of sexual intercourse | r=-0.14  p<0.001 | r=0.02  p<0.001 | r=-0.08  p<0.001 |  |  |  |

Note. We used continuous measures where possible (rather than the categorised measures used in the main analysis). Continuous measures were associated using Pearson’s correlation. Binary variables were associated with continuous measures using independent samples t-test. Binary measures were associated with each other using chi-squared test. Below the diagonal is the Pearson’s correlation coefficient and p-value for correlations, the group means and t-test p-value for t-tests and chi-squared statistic and p-value for chi-squared tests. Above the diagonal is the number of individuals available for each comparison.

**Supplementary Table S3. Summary of female characteristics comparing planners and non-planners**

|  | Non-Planners  (N = 15,600) | Planners  (N = 69,788) |  |
| --- | --- | --- | --- |
|  | **Mean /%** | **Mean /%** | **P-value** |
| Age (years) | 28.98 | 30.55 | <0.001 |
| University education | 46% | 67% | <0.001 |
| BMI (kg/m2) | 23.97 | 24.08 | 0.005 |
| Alcohol consumption |  |  | <0.001 |
| *Never* | 7.6% | 7.3% |  |
| *Daily* | 0.3% | 0.2% |  |
| Binge drinking |  |  | <0.001 |
| *Never* | 29% | 35% |  |
| *Several times per week* | 1.8% | 0.6% |  |
| Ever smokers | 60% | 48% | <0.001 |
| Smoking heaviness (cigarettes per day) | 12.38 | 10.93 | <0.001 |
| Caffeine consumption (mg per day) | 149.32 | 139.16 | <0.001 |
| Age at first birth (years) | 25.78 | 27.62 | <0.001 |
| Number of children (N children) | 2.61 | 2.52 | <0.001 |

**Supplementary Table S4. Sensitivity analysis in females including non-planners in time to conception observational analyses compared with primary analysis with planners only**

|  | **Planners only** | | | **Including non-planners** | | |
| --- | --- | --- | --- | --- | --- | --- |
| **Exposure** | **N** | **Beta (95% CI)** | **P-value** | **N** | **Beta (95% CI)** | **P-value** |
| Alcohol Consumption | 63085 | -0.019 (-0.045, 0.007) | 0.148 | 75915 | -0.038 (-0.060, -0.017) | <0.001 |
| Binge Drinking | 62501 | 0.030 (-0.001, 0.061) | 0.061 | 75101 | -0.022 (-0.049, 0.004) | 0.092 |
| Caffeine consumption | 58350 | 0.010 (-0.014, 0.034) | 0.402 | 70622 | -0.003 (-0.023, 0.017) | 0.779 |
| BMI | 64593 | 0.059 ( 0.053, 0.066) | <0.001 | 78164 | 0.052 (0.046, 0.057) | <0.001 |
| Smoking Initiation | 65395 | 0.094 ( 0.039, 0.149) | <0.001 | 78835 | 0.024 (-0.022, 0.071) | 0.307 |
| Smoking Heaviness | 10867 | 0.028 ( 0.015, 0.040) | <0.001 | 15091 | 0.008 (-0.001, 0.017) | 0.087 |

Note. All estimates are adjusted for birth year and educational attainment. Non-planners were given the median time to conception (2 months). Results are for both genotyped and non-genotyped individuals.

**Supplementary Table S5. Sensitivity analysis in females including non-planners in time to conception Mendelian randomisation analyses compared with planners only**

|  | **Planners only** | | | **Non-planners included** | | |
| --- | --- | --- | --- | --- | --- | --- |
| **Exposure** | **N** | **Beta (95% CI)** | **P-value** | **N** | **Beta (95% CI)** | **P-value** |
| Alcohol consumption | 48,532 | 0.300 (-0.491, 1.091) | 0.457 | 57,954 | -1.991 (-2.904, -1.078) | <0.001 |
| Caffeine consumption | 44,628 | 0.290 (-0.162, 0.742) | 0.209 | 53,321 | -0.182 (-0.634, 0.270) | 0.430 |
| BMI | 49,310 | 0.049 ( 0.020, 0.077) | <0.001 | 58,863 | 0.878 (0.776, 0.981) | <0.001 |
| Smoking Initiation | 49,925 | 0.462 (-0.054, 0.978) | 0.080 | 59,612 | 0.184 (-0.040, 0.409) | 0.108 |
| CPD | 8,374 | 0.046 (-0.084, 0.176) | 0.491 | 11,451 | 0.450 (-0.104, 1.004) | 0.112 |

Note. Individual-level MR results in the females. Betas can be interpreted as risk difference.

**Supplementary Table S6. Frequency of sexual intercourse in the four weeks prior to conception.**

|  | Females | | |  |
| --- | --- | --- | --- | --- |
|  | Full Sample |  | Genotyped Sample | |
|  | **N** | **Mean (SD)/%** | **N** | **Mean (SD)/%** |
| *Never* | 528 | 0.63% | 374 | 0.58% |
| *Less than 1-2 times every 2 weeks* | 3,390 | 4.02% | 2,615 | 4.09% |
| *1-2 times every two weeks* | 11,587 | 13.72% | 8,893 | 13.91% |
| *1-2 times a week* | 30,297 | 35.89% | 23,091 | 36.11% |
| *3-4 times a week* | 28,224 | 33.43% | 21,300 | 33.31% |
| *5-6 times a week* | 6,872 | 8.14% | 5,154 | 8.06% |
| *Every day* | 3,529 | 4.18% | 2,527 | 3.95% |
|  | **Males** |  |  |  |
|  | Full Sample |  | Genotyped Sample | |
|  | **N** | **Mean (SD)/%** | **N** | **Mean (SD)/%** |
| *Never* | 349 | 0.51% | 218 | 0.48% |
| *Less than 1-2 times every 2 weeks* | 2,541 | 3.75% | 1,756 | 3.86% |
| *1-2 times every two weeks* | 9,313 | 13.73% | 6,332 | 13.93% |
| *1-2 times a week* | 24,596 | 36.27% | 16,558 | 36.41% |
| *3-4 times a week* | 22,837 | 33.68% | 15,320 | 33.69% |
| *5-6 times a week* | 5,442 | 8.02% | 3,565 | 7.84% |
| *Every day* | 2,737 | 4.04% | 1,722 | 3.79% |

**Supplementary Table S7. Observational associations between health behaviours and fertility/reproductive outcomes in females (full sample)**

|  |  |  | **Unadjusted** |  | **Adjusted for birth year and education** |  | **Adjusted for birth year, education and ADHD** |  |
| --- | --- | --- | --- | --- | --- | --- | --- | --- |
| **Exposure** | **Outcome** | **N** | **Beta (95% CI)** | **P-value** | **Beta (95% CI)** | **P-value** | **Beta (95% CI)** | **P-value** |
| Alcohol Frequency | Age at First Birth | 80706 | 0.888 ( 0.859, 0.916) | 0.00e+00 | 0.479 ( 0.455, 0.503) | 0.00e+00 | 0.504 ( 0.473, 0.535) | 1.14e-221 |
| Alcohol Frequency | Number of Children | 80706 | -0.095 (-0.100, -0.089) | 2.68e-252 | -0.091 (-0.096, -0.085) | 1.38e-212 | -0.087 (-0.094, -0.080) | 5.70e-119 |
| Alcohol Frequency | Sex Frequency | 79157 | 0.043 ( 0.036, 0.050) | 1.13e-32 | 0.072 ( 0.064, 0.079) | 1.16e-83 | 0.073 ( 0.064, 0.082) | 4.58e-53 |
| Alcohol Frequency | Time to Conception | 63085 | -0.021 (-0.046, 0.004) | 0.094 | -0.019 (-0.045, 0.007) | 0.148 | -0.002 (-0.036, 0.032) | 0.902 |
| Binge Drinking | Age at First Birth | 79806 | -0.086 (-0.121, -0.051) | 1.46e-06 | 0.317 ( 0.289, 0.346) | 3.70e-104 | 0.397 ( 0.359, 0.435) | 2.43e-94 |
| Binge Drinking | Number of Children | 79806 | -0.115 (-0.121, -0.108) | 3.28e-254 | -0.111 (-0.118, -0.104) | 1.73e-222 | -0.115 (-0.124, -0.106) | 6.02e-142 |
| Binge Drinking | Sex Frequency | 78306 | 0.100 ( 0.092, 0.109) | 3.72e-117 | 0.067 ( 0.058, 0.075) | 5.63e-51 | 0.063 ( 0.052, 0.075) | 4.59e-28 |
| Binge Drinking | Time to Conception | 62501 | -0.011 (-0.041, 0.019) | 0.476 | 0.030 (-0.001, 0.061) | 0.061 | 0.035 (-0.006, 0.076) | 0.096 |
| Caffeine Consumption | Age at First Birth | 76255 | 0.311 ( 0.284, 0.338) | 9.44e-113 | -0.008 (-0.030, 0.015) | 0.498 | 0.033 ( 0.004, 0.063) | 0.028 |
| Caffeine Consumption | Number of Children | 76255 | 0.002 (-0.003, 0.007) | 0.431 | -0.005 (-0.011, 0.00005) | 0.052 | -0.007 (-0.014, -0.00030) | 0.041 |
| Caffeine Consumption | Sex Frequency | 74735 | -0.009 (-0.016, -0.003) | 0.005 | 0.020 ( 0.013, 0.026) | 8.41e-09 | 0.020 ( 0.011, 0.028) | 1.25e-05 |
| Caffeine Consumption | Time to Conception | 58350 | 0.052 ( 0.029, 0.076) | 9.60e-06 | 0.010 (-0.014, 0.034) | 0.402 | 0.008 (-0.024, 0.040) | 0.629 |
| BMI | Age at First Birth | 84057 | -0.042 (-0.049, -0.035) | 4.02e-30 | -0.016 (-0.022, -0.010) | 6.48e-08 | -0.012 (-0.020, -0.004) | 0.003 |
| BMI | Number of Children | 84057 | -0.00100 (-0.002, 0.00040) | 0.168 | -0.004 (-0.005, -0.002) | 1.82e-07 | -0.006 (-0.008, -0.004) | 6.27e-09 |
| BMI | Sex Frequency | 82725 | -0.007 (-0.009, -0.005) | 6.96e-15 | -0.007 (-0.008, -0.005) | 4.57e-13 | -0.006 (-0.009, -0.004) | 2.11e-07 |
| BMI | Time to Conception | 64593 | 0.068 ( 0.061, 0.074) | 1.69e-100 | 0.059 ( 0.053, 0.066) | 5.29e-73 | 0.053 ( 0.044, 0.062) | 1.01e-32 |
| Smoking Initiation | Age at First Birth | 83899 | -1.060 (-1.122, -0.998) | 3.18e-246 | -0.161 (-0.213, -0.110) | 9.66e-10 | -0.010 (-0.077, 0.058) | 0.783 |
| Smoking Initiation | Number of Children | 83899 | -0.075 (-0.087, -0.063) | 1.34e-34 | -0.098 (-0.111, -0.086) | 7.97e-54 | -0.111 (-0.127, -0.094) | 7.65e-41 |
| Smoking Initiation | Sex Frequency | 82188 | 0.040 ( 0.025, 0.055) | 2.97e-07 | -0.015 (-0.031, 0.00080) | 0.063 | 0.002 (-0.019, 0.022) | 0.868 |
| Smoking Initiation | Time to Conception | 65395 | 0.137 ( 0.084, 0.190) | 3.21e-07 | 0.094 ( 0.039, 0.149) | 7.63e-04 | 0.117 ( 0.045, 0.190) | 0.002 |
| Smoking Heaviness | Age at First Birth | 16420 | -0.084 (-0.097, -0.072) | 3.57e-39 | -0.007 (-0.018, 0.004) | 0.229 | 0.009 (-0.007, 0.025) | 0.279 |
| Smoking Heaviness | Number of Children | 16420 | -0.002 (-0.005, -0.00002) | 0.048 | -0.008 (-0.010, -0.005) | 7.28e-09 | -0.011 (-0.014, -0.007) | 1.00e-08 |
| Smoking Heaviness | Sex Frequency | 16040 | 0.013 ( 0.010, 0.016) | 7.63e-15 | 0.007 ( 0.004, 0.011) | 1.21e-05 | 0.005 ( 0.00020, 0.010) | 0.040 |
| Smoking Heaviness | Time to Conception | 10867 | 0.031 ( 0.020, 0.043) | 1.92e-07 | 0.028 ( 0.015, 0.040) | 1.33e-05 | 0.029 ( 0.011, 0.047) | 0.002 |
| **Exposure** | **Outcome** | **N** | **OR (95% CI)** | **P-value** | **OR (95% CI)** | **P-value** | **OR (95% CI)** | **P-value** |
| Alcohol Frequency | Subfertile | 63085 | 0.989 (0.966, 1.014) | 0.386 | 0.988 (0.963, 1.013) | 0.356 | 1.005 (0.973, 1.038) | 0.776 |
| Alcohol Frequency | Highly Fertile | 79508 | 1.016 (1.001, 1.031) | 0.042 | 1.012 (0.996, 1.028) | 0.134 | 1.012 (0.991, 1.033) | 0.260 |
| Alcohol Frequency | Infertility Treatment | 79869 | 0.908 (0.888, 0.928) | 2.29e-17 | 0.875 (0.854, 0.895) | 4.59e-29 | 0.879 (0.853, 0.906) | 1.07e-16 |
| Alcohol Frequency | Miscarriage | 54337 | 1.000 (0.983, 1.017) | 0.971 | 0.990 (0.973, 1.008) | 0.268 | 0.986 (0.963, 1.009) | 0.238 |
| Binge Drinking | Subfertile | 62501 | 0.988 (0.960, 1.018) | 0.446 | 1.020 (0.990, 1.052) | 0.197 | 1.028 (0.988, 1.070) | 0.177 |
| Binge Drinking | Highly Fertile | 78659 | 1.019 (1.000, 1.037) | 0.045 | 1.000 (0.981, 1.019) | 1.000 | 0.995 (0.970, 1.020) | 0.700 |
| Binge Drinking | Infertility Treatment | 78994 | 0.762 (0.740, 0.784) | 1.43e-76 | 0.822 (0.798, 0.847) | 6.09e-38 | 0.825 (0.793, 0.857) | 3.06e-22 |
| Binge Drinking | Miscarriage | 53752 | 0.958 (0.938, 0.979) | 1.03e-04 | 0.970 (0.949, 0.992) | 0.008 | 0.983 (0.954, 1.013) | 0.257 |
| Caffeine Consumption | Subfertile | 58350 | 1.051 (1.028, 1.076) | 1.78e-05 | 1.013 (0.989, 1.037) | 0.295 | 1.004 (0.973, 1.035) | 0.810 |
| Caffeine Consumption | Highly Fertile | 75096 | 0.986 (0.973, 1.000) | 0.051 | 1.008 (0.994, 1.023) | 0.265 | 1.011 (0.991, 1.031) | 0.298 |
| Caffeine Consumption | Infertility Treatment | 75075 | 1.020 (0.999, 1.041) | 0.064 | 0.951 (0.931, 0.972) | 4.72e-06 | 0.956 (0.930, 0.984) | 0.002 |
| Caffeine Consumption | Miscarriage | 51839 | 1.015 (0.999, 1.031) | 0.066 | 1.002 (0.986, 1.019) | 0.793 | 1.011 (0.989, 1.034) | 0.315 |
| BMI | Subfertile | 64593 | 1.051 (1.045, 1.057) | 5.65e-71 | 1.046 (1.040, 1.052) | 8.59e-53 | 1.043 (1.035, 1.051) | 1.05e-26 |
| BMI | Highly Fertile | 83112 | 0.982 (0.979, 0.986) | 1.23e-19 | 0.987 (0.983, 0.991) | 2.04e-10 | 0.987 (0.982, 0.993) | 6.63e-06 |
| BMI | Infertility Treatment | 83090 | 1.036 (1.031, 1.041) | 8.31e-44 | 1.032 (1.026, 1.037) | 6.12e-30 | 1.033 (1.026, 1.041) | 1.05e-18 |
| BMI | Miscarriage | 56890 | 1.008 (1.004, 1.012) | 1.28e-04 | 1.008 (1.004, 1.013) | 1.43e-04 | 1.010 (1.005, 1.016) | 5.62e-04 |
| Smoking Initiation | Subfertile | 65395 | 1.112 (1.057, 1.171) | 5.08e-05 | 1.074 (1.018, 1.134) | 0.009 | 1.087 (1.013, 1.166) | 0.021 |
| Smoking Initiation | Highly Fertile | 82567 | 0.955 (0.925, 0.986) | 0.005 | 0.969 (0.937, 1.002) | 0.068 | 0.943 (0.902, 0.987) | 0.011 |
| Smoking Initiation | Infertility Treatment | 82956 | 0.932 (0.889, 0.977) | 0.003 | 0.958 (0.912, 1.007) | 0.093 | 0.955 (0.895, 1.020) | 0.170 |
| Smoking Initiation | Miscarriage | 56454 | 0.958 (0.924, 0.993) | 0.019 | 0.977 (0.940, 1.015) | 0.223 | 0.983 (0.934, 1.034) | 0.502 |
| Smoking Heaviness | Subfertile | 10867 | 1.029 (1.019, 1.039) | 8.90e-09 | 1.028 (1.017, 1.038) | 1.73e-07 | 1.029 (1.014, 1.044) | 7.96e-05 |
| Smoking Heaviness | Highly Fertile | 16082 | 0.998 (0.992, 1.005) | 0.602 | 1.003 (0.996, 1.009) | 0.453 | 1.001 (0.991, 1.011) | 0.877 |
| Smoking Heaviness | Infertility Treatment | 16072 | 1.006 (0.996, 1.015) | 0.256 | 1.006 (0.996, 1.017) | 0.219 | 1.007 (0.992, 1.022) | 0.379 |
| Smoking Heaviness | Miscarriage | 10959 | 1.006 (0.999, 1.013) | 0.089 | 1.004 (0.997, 1.012) | 0.277 | 1.003 (0.992, 1.014) | 0.566 |

**Supplementary Table S8. Observational associations between health behaviours and fertility/reproductive outcomes in males (full sample)**

|  |  |  | **Unadjusted** |  | **Adjusted for birth year and education** |  | **Adjusted for birth year, education and ADHD** |  |
| --- | --- | --- | --- | --- | --- | --- | --- | --- |
| **Exposure** | **Outcome** | **N** | **Beta (95% CI)** | **P-value** | **Beta (95% CI)** | **P-value** | **Beta (95% CI)** | **P-Value** |
| Alcohol Frequency | Age at First Birth | 64516 | 0.624 ( 0.591, 0.658) | 3.74e-286 | 0.322 ( 0.292, 0.352) | 2.02e-99 | 0.218 ( 0.179, 0.257) | 1.12e-27 |
| Alcohol Frequency | Number of Children | 64516 | -0.051 (-0.057, -0.045) | 8.00e-63 | -0.055 (-0.062, -0.049) | 1.34e-64 | -0.050 (-0.059, -0.042) | 1.98e-31 |
| Alcohol Frequency | Sex Frequency | 62454 | 0.015 ( 0.007, 0.023) | 1.06e-04 | 0.041 ( 0.033, 0.049) | 9.53e-24 | 0.054 ( 0.043, 0.065) | 1.74e-21 |
| Alcohol Frequency | Time to Conception | 50686 | 0.010 (-0.016, 0.037) | 0.454 | 0.022 (-0.006, 0.051) | 0.128 | 0.021 (-0.018, 0.060) | 0.286 |
| Binge Drinking | Age at First Birth | 31194 | -0.110 (-0.169, -0.051) | 2.80e-04 | 0.283 ( 0.235, 0.331) | 9.48e-31 | 0.295 ( 0.246, 0.343) | 1.31e-32 |
| Binge Drinking | Number of Children | 31194 | -0.092 (-0.102, -0.082) | 4.49e-73 | -0.081 (-0.092, -0.071) | 1.10e-52 | -0.083 (-0.093, -0.072) | 6.94e-54 |
| Binge Drinking | Sex Frequency | 30358 | 0.099 ( 0.086, 0.112) | 6.79e-48 | 0.077 ( 0.063, 0.091) | 4.24e-28 | 0.080 ( 0.066, 0.094) | 6.70e-30 |
| Binge Drinking | Time to Conception | 24864 | -0.006 (-0.052, 0.040) | 0.793 | 0.022 (-0.026, 0.071) | 0.361 | 0.020 (-0.028, 0.069) | 0.408 |
| Caffeine Consumption | Age at First Birth | 29940 | 0.196 ( 0.151, 0.241) | 2.18e-17 | -0.103 (-0.140, -0.066) | 3.98e-08 | -0.099 (-0.136, -0.062) | 1.37e-07 |
| Caffeine Consumption | Number of Children | 29940 | 0.017 ( 0.010, 0.025) | 8.28e-06 | 0.011 ( 0.003, 0.019) | 0.007 | 0.011 ( 0.003, 0.019) | 0.007 |
| Caffeine Consumption | Sex Frequency | 29122 | -0.024 (-0.034, -0.014) | 4.58e-06 | -0.004 (-0.015, 0.007) | 0.452 | -0.004 (-0.014, 0.007) | 0.490 |
| Caffeine Consumption | Time to Conception | 23896 | 0.048 ( 0.013, 0.083) | 0.007 | 0.024 (-0.013, 0.060) | 0.202 | 0.022 (-0.014, 0.059) | 0.233 |
| BMI | Age at First Birth | 68002 | 0.038 ( 0.027, 0.049) | 2.76e-11 | 0.025 ( 0.016, 0.035) | 2.64e-07 | 0.00050 (-0.012, 0.013) | 0.940 |
| BMI | Number of Children | 68002 | -0.003 (-0.005, -0.0006) | 0.011 | -0.006 (-0.008, -0.004) | 2.62e-08 | -0.006 (-0.008, -0.003) | 1.25e-04 |
| BMI | Sex Frequency | 66299 | -0.008 (-0.011, -0.006) | 2.82e-10 | -0.008 (-0.010, -0.005) | 1.12e-08 | -0.006 (-0.010, -0.003) | 8.60e-04 |
| BMI | Time to Conception | 53718 | 0.053 ( 0.045, 0.062) | 1.28e-32 | 0.043 ( 0.033, 0.052) | 1.85e-19 | 0.044 ( 0.031, 0.057) | 4.71e-11 |
| Smoking Initiation | Age at First Birth | 66159 | -0.676 (-0.752, -0.601) | 8.42e-69 | -0.209 (-0.275, -0.143) | 5.44e-10 | -0.099 (-0.188, -0.010) | 0.030 |
| Smoking Initiation | Number of Children | 66159 | -0.016 (-0.029, -0.002) | 0.022 | -0.024 (-0.038, -0.010) | 9.39e-04 | -0.030 (-0.049, -0.010) | 0.003 |
| Smoking Initiation | Sex Frequency | 63993 | 0.011 (-0.006, 0.028) | 0.188 | -0.016 (-0.034, 0.002) | 0.080 | 0.004 (-0.021, 0.030) | 0.742 |
| Smoking Initiation | Time to Conception | 51956 | 0.115 ( 0.056, 0.174) | 1.28e-04 | 0.032 (-0.031, 0.094) | 0.319 | -0.011 (-0.099, 0.077) | 0.799 |
| Smoking Heaviness | Age at First Birth | 12691 | -0.037 (-0.052, -0.023) | 8.26e-07 | -0.008 (-0.021, 0.005) | 0.237 | 0.006 (-0.013, 0.026) | 0.526 |
| Smoking Heaviness | Number of Children | 12691 | -0.004 (-0.006, -0.0008) | 0.010 | -0.005 (-0.007, -0.002) | 0.002 | -0.006 (-0.010, -0.002) | 0.007 |
| Smoking Heaviness | Sex Frequency | 12157 | 0.009 ( 0.006, 0.012) | 9.35e-08 | 0.006 ( 0.002, 0.009) | 7.89e-04 | 0.003 (-0.003, 0.008) | 0.350 |
| Smoking Heaviness | Time to Conception | 9112 | 0.025 ( 0.014, 0.037) | 2.70e-05 | 0.020 ( 0.008, 0.033) | 0.002 | 0.021 ( 0.001, 0.041) | 0.036 |
| **Exposure** | **Outcome** | **N** | **OR (95% CI)** | **P-value** | **OR (95% CI)** | **P-value** | **OR (95% CI)** | **P-Value** |
| Alcohol Frequency | Subfertile | 50686 | 1.017 (0.991, 1.044) | 0.208 | 1.021 (0.993, 1.050) | 0.139 | 1.021 (0.982, 1.062) | 0.301 |
| Alcohol Frequency | Highly Fertile | 63249 | 1.017 (1.000, 1.034) | 0.044 | 1.011 (0.993, 1.029) | 0.224 | 1.015 (0.990, 1.041) | 0.231 |
| Binge Drinking | Subfertile | 24864 | 1.017 (0.970, 1.066) | 0.482 | 1.047 (0.997, 1.100) | 0.063 | 1.044 (0.994, 1.097) | 0.085 |
| Binge Drinking | Highly Fertile | 30713 | 1.028 (0.999, 1.058) | 0.056 | 1.010 (0.980, 1.041) | 0.518 | 1.011 (0.981, 1.042) | 0.485 |
| Caffeine Consumption | Subfertile | 23896 | 1.031 (0.994, 1.069) | 0.107 | 1.010 (0.972, 1.049) | 0.617 | 1.006 (0.968, 1.045) | 0.763 |
| Caffeine Consumption | Highly Fertile | 29481 | 0.987 (0.966, 1.009) | 0.249 | 1.002 (0.979, 1.025) | 0.892 | 1.002 (0.979, 1.025) | 0.876 |
| BMI | Subfertile | 53718 | 1.041 (1.032, 1.050) | 1.76e-21 | 1.034 (1.025, 1.043) | 2.07e-13 | 1.039 (1.026, 1.052) | 2.94e-09 |
| BMI | Highly Fertile | 67119 | 0.976 (0.971, 0.982) | 1.04e-17 | 0.982 (0.976, 0.987) | 3.63e-10 | 0.984 (0.976, 0.992) | 1.71e-04 |
| Smoking Initiation | Subfertile | 51956 | 1.072 (1.011, 1.136) | 0.019 | 0.994 (0.934, 1.058) | 0.854 | 0.987 (0.901, 1.080) | 0.776 |
| Smoking Initiation | Highly Fertile | 64840 | 0.958 (0.924, 0.994) | 0.021 | 0.990 (0.952, 1.029) | 0.596 | 1.018 (0.963, 1.077) | 0.529 |
| Smoking Heaviness | Subfertile | 9112 | 1.024 (1.014, 1.034) | 2.02e-06 | 1.020 (1.009, 1.031) | 2.76e-04 | 1.021 (1.004, 1.039) | 0.014 |
| Smoking Heaviness | Highly Fertile | 12394 | 0.999 (0.992, 1.006) | 0.790 | 1.001 (0.994, 1.008) | 0.795 | 1.000 (0.989, 1.011) | 0.972 |

**Supplementary Table S9. Observational associations between health behaviours and fertility/reproductive outcomes in genotyped females**

|  |  |  | **Unadjusted** |  | **Adjusted for birth year and education** |  | **Adjusted for birth year, education and ADHD** |  |
| --- | --- | --- | --- | --- | --- | --- | --- | --- |
| **Exposure** | **Outcome** | **N** | **Beta (95% CI)** | **P-value** | **Beta (95% CI)** | **P-value** | **Beta (95% CI)** | **P-value** |
| Alcohol Frequency | Age at First Birth | 61721 | 0.906 ( 0.874, 0.938) | 0.00e+00 | 0.495 ( 0.468, 0.522) | 3.92e-280 | 0.508 ( 0.473, 0.543) | 1.07e-178 |
| Alcohol Frequency | Number of Children | 61721 | -0.095 (-0.101, -0.088) | 6.50e-196 | -0.092 (-0.098, -0.085) | 1.29e-169 | -0.088 (-0.096, -0.080) | 6.98e-96 |
| Alcohol Frequency | Sex Frequency | 60611 | 0.041 ( 0.033, 0.050) | 6.15e-24 | 0.069 ( 0.061, 0.078) | 7.44e-61 | 0.069 ( 0.059, 0.080) | 1.49e-37 |
| Alcohol Frequency | Time to Conception | 48647 | -0.028 (-0.056, -0.00020) | 0.048 | -0.025 (-0.054, 0.004) | 0.092 | -0.021 (-0.059, 0.017) | 0.280 |
| Binge Drinking | Age at First Birth | 61105 | -0.071 (-0.111, -0.031) | 4.49e-04 | 0.339 ( 0.306, 0.371) | 8.64e-92 | 0.411 ( 0.369, 0.453) | 3.50e-80 |
| Binge Drinking | Number of Children | 61105 | -0.115 (-0.123, -0.108) | 3.83e-199 | -0.112 (-0.120, -0.104) | 3.56e-176 | -0.116 (-0.126, -0.106) | 3.65e-113 |
| Binge Drinking | Sex Frequency | 60024 | 0.101 ( 0.091, 0.110) | 7.90e-91 | 0.067 ( 0.057, 0.077) | 6.56e-40 | 0.062 ( 0.049, 0.075) | 3.52e-21 |
| Binge Drinking | Time to Conception | 48242 | -0.028 (-0.062, 0.007) | 0.118 | 0.013 (-0.023, 0.048) | 0.475 | 0.019 (-0.027, 0.066) | 0.414 |
| Caffeine Consumption | Age at First Birth | 57881 | 0.328 ( 0.297, 0.358) | 1.94e-96 | 0.016 (-0.010, 0.041) | 0.238 | 0.049 ( 0.015, 0.083) | 0.004 |
| Caffeine Consumption | Number of Children | 57881 | 0.00080 (-0.005, 0.007) | 0.796 | -0.007 (-0.013, -0.00080) | 0.026 | -0.008 (-0.016, -0.00050) | 0.038 |
| Caffeine Consumption | Sex Frequency | 56820 | -0.010 (-0.018, -0.003) | 0.007 | 0.019 ( 0.011, 0.027) | 1.28e-06 | 0.020 ( 0.010, 0.030) | 8.96e-05 |
| Caffeine Consumption | Time to Conception | 44742 | 0.054 ( 0.027, 0.080) | 7.26e-05 | 0.012 (-0.015, 0.040) | 0.377 | 0.016 (-0.020, 0.052) | 0.388 |
| BMI | Age at First Birth | 63627 | -0.043 (-0.052, -0.035) | 4.58e-25 | -0.011 (-0.018, -0.004) | 0.002 | -0.010 (-0.019, -0.00070) | 0.034 |
| BMI | Number of Children | 63627 | -0.001 (-0.003, 0.00020) | 0.096 | -0.004 (-0.006, -0.002) | 7.31e-07 | -0.006 (-0.008, -0.004) | 7.52e-08 |
| BMI | Sex Frequency | 62714 | -0.005 (-0.007, -0.003) | 1.78e-06 | -0.005 (-0.007, -0.003) | 4.09e-06 | -0.005 (-0.008, -0.002) | 3.70e-04 |
| BMI | Time to Conception | 49425 | 0.070 ( 0.062, 0.077) | 5.34e-81 | 0.060 ( 0.053, 0.067) | 5.10e-57 | 0.053 ( 0.043, 0.063) | 2.39e-25 |
| Smoking Initiation | Age at First Birth | 63500 | -1.033 (-1.103, -0.962) | 2.09e-180 | -0.094 (-0.153, -0.035) | 0.002 | 0.022 (-0.055, 0.098) | 0.579 |
| Smoking Initiation | Number of Children | 63500 | -0.072 (-0.086, -0.058) | 2.29e-25 | -0.096 (-0.110, -0.082) | 3.99e-40 | -0.108 (-0.127, -0.090) | 2.29e-31 |
| Smoking Initiation | Sex Frequency | 62327 | 0.039 ( 0.021, 0.056) | 1.40e-05 | -0.015 (-0.033, 0.003) | 0.104 | -0.002 (-0.025, 0.021) | 0.893 |
| Smoking Initiation | Time to Conception | 50043 | 0.138 ( 0.078, 0.198) | 7.10e-06 | 0.089 ( 0.026, 0.151) | 0.005 | 0.117 ( 0.035, 0.199) | 0.005 |
| Smoking Heaviness | Age at First Birth | 12610 | -0.089 (-0.103, -0.074) | 1.59e-33 | -0.010 (-0.023, 0.002) | 0.111 | 0.009 (-0.009, 0.027) | 0.338 |
| Smoking Heaviness | Number of Children | 12610 | -0.002 (-0.005, 0.00050) | 0.101 | -0.007 (-0.010, -0.004) | 2.42e-06 | -0.009 (-0.013, -0.005) | 5.48e-06 |
| Smoking Heaviness | Sex Frequency | 12337 | 0.012 ( 0.008, 0.015) | 3.70e-10 | 0.006 ( 0.003, 0.010) | 9.92e-04 | 0.006 ( 0.00002, 0.011) | 0.049 |
| Smoking Heaviness | Time to Conception | 8440 | 0.031 ( 0.017, 0.044) | 7.62e-06 | 0.028 ( 0.014, 0.042) | 1.30e-04 | 0.029 ( 0.009, 0.049) | 0.005 |
| **Exposure** | **Outcome** | **N** | **OR (95% CI)** | **P-value** | **OR (95% CI)** | **P-value** | **OR (95% CI)** | **P-value** |
| Alcohol Frequency | Subfertile | 48647 | 0.985 (0.959, 1.013) | 0.296 | 0.984 (0.956, 1.013) | 0.282 | 0.994 (0.959, 1.032) | 0.768 |
| Alcohol Frequency | Highly Fertile | 60852 | 1.018 (1.000, 1.035) | 0.045 | 1.014 (0.996, 1.033) | 0.122 | 1.022 (0.998, 1.046) | 0.077 |
| Alcohol Frequency | Infertility Treatment | 61117 | 0.900 (0.877, 0.923) | 5.46e-16 | 0.866 (0.843, 0.889) | 5.04e-26 | 0.869 (0.839, 0.899) | 1.03e-15 |
| Alcohol Frequency | Miscarriage | 41755 | 0.988 (0.970, 1.008) | 0.238 | 0.978 (0.958, 0.997) | 0.027 | 0.975 (0.950, 1.001) | 0.063 |
| Binge Drinking | Subfertile | 48242 | 0.977 (0.944, 1.011) | 0.179 | 1.010 (0.975, 1.046) | 0.575 | 1.024 (0.979, 1.071) | 0.303 |
| Binge Drinking | Highly Fertile | 60266 | 1.028 (1.007, 1.050) | 0.008 | 1.010 (0.988, 1.032) | 0.382 | 1.008 (0.979, 1.037) | 0.605 |
| Binge Drinking | Infertility Treatment | 60516 | 0.756 (0.732, 0.782) | 1.74e-62 | 0.816 (0.789, 0.844) | 6.84e-32 | 0.817 (0.781, 0.853) | 2.25e-19 |
| Binge Drinking | Miscarriage | 41353 | 0.944 (0.921, 0.968) | 5.42e-06 | 0.955 (0.931, 0.980) | 4.61e-04 | 0.970 (0.937, 1.003) | 0.074 |
| Caffeine Consumption | Subfertile | 44742 | 1.055 (1.028, 1.083) | 6.27e-05 | 1.016 (0.989, 1.044) | 0.242 | 1.012 (0.978, 1.048) | 0.493 |
| Caffeine Consumption | Highly Fertile | 57068 | 0.984 (0.968, 1.000) | 0.045 | 1.005 (0.988, 1.022) | 0.571 | 1.006 (0.984, 1.029) | 0.583 |
| Caffeine Consumption | Infertility Treatment | 57029 | 1.026 (1.003, 1.051) | 0.030 | 0.957 (0.933, 0.980) | 3.78e-04 | 0.968 (0.938, 0.999) | 0.046 |
| Caffeine Consumption | Miscarriage | 39520 | 1.013 (0.995, 1.032) | 0.150 | 1.001 (0.982, 1.020) | 0.936 | 1.011 (0.986, 1.036) | 0.396 |
| BMI | Subfertile | 49425 | 1.052 (1.045, 1.059) | 2.61e-56 | 1.046 (1.039, 1.053) | 1.40e-40 | 1.042 (1.033, 1.052) | 1.70e-20 |
| BMI | Highly Fertile | 62991 | 0.982 (0.978, 0.986) | 8.76e-16 | 0.987 (0.983, 0.992) | 3.85e-08 | 0.989 (0.983, 0.995) | 5.36e-04 |
| BMI | Infertility Treatment | 62958 | 1.036 (1.030, 1.042) | 6.14e-34 | 1.031 (1.025, 1.037) | 1.37e-22 | 1.032 (1.023, 1.040) | 7.13e-14 |
| BMI | Miscarriage | 43297 | 1.007 (1.002, 1.011) | 0.006 | 1.006 (1.001, 1.011) | 0.011 | 1.006 (1.000, 1.013) | 0.062 |
| Smoking Initiation | Subfertile | 50043 | 1.115 (1.051, 1.182) | 2.96e-04 | 1.077 (1.012, 1.145) | 0.019 | 1.102 (1.018, 1.193) | 0.017 |
| Smoking Initiation | Highly Fertile | 62588 | 0.951 (0.916, 0.986) | 0.007 | 0.971 (0.934, 1.009) | 0.136 | 0.942 (0.895, 0.991) | 0.022 |
| Smoking Initiation | Infertility Treatment | 62868 | 0.951 (0.901, 1.003) | 0.066 | 0.972 (0.918, 1.029) | 0.323 | 0.969 (0.900, 1.044) | 0.406 |
| Smoking Initiation | Miscarriage | 42986 | 0.950 (0.912, 0.991) | 0.016 | 0.964 (0.923, 1.007) | 0.098 | 0.975 (0.920, 1.033) | 0.390 |
| Smoking Heaviness | Subfertile | 8440 | 1.028 (1.017, 1.039) | 9.63e-07 | 1.028 (1.016, 1.040) | 4.17e-06 | 1.028 (1.012, 1.045) | 5.73e-04 |
| Smoking Heaviness | Highly Fertile | 12359 | 0.998 (0.991, 1.005) | 0.621 | 1.001 (0.994, 1.009) | 0.713 | 0.997 (0.985, 1.008) | 0.559 |
| Smoking Heaviness | Infertility Treatment | 12355 | 1.004 (0.993, 1.015) | 0.511 | 1.006 (0.995, 1.018) | 0.293 | 1.010 (0.994, 1.027) | 0.217 |
| Smoking Heaviness | Miscarriage | 8425 | 1.005 (0.998, 1.014) | 0.179 | 1.004 (0.995, 1.012) | 0.401 | 1.006 (0.993, 1.018) | 0.385 |

**Supplementary Table S10. Observational associations between health behaviours and fertility/reproductive outcomes in the genotyped males**

|  |  |  | **Unadjusted** |  | **Adjusted for birthyear and education** |  | **Adjusted for birthyear, education and ADHD** |  |
| --- | --- | --- | --- | --- | --- | --- | --- | --- |
| **Exposure** | **Outcome** | **N** | **Beta (95% CI)** | **P-value** | **Beta (95% CI)** | **P-value** | **Beta (95% CI)** | **P-value** |
| Alcohol Frequency | Age at First Birth | 43211 | 0.649 ( 0.608, 0.690) | 4.37e-209 | 0.324 ( 0.288, 0.360) | 2.86e-69 | 0.215 ( 0.168, 0.262) | 2.92e-19 |
| Alcohol Frequency | Number of Children | 43211 | -0.046 (-0.053, -0.038) | 9.02e-35 | -0.049 (-0.057, -0.041) | 2.41e-35 | -0.045 (-0.055, -0.035) | 5.57e-18 |
| Alcohol Frequency | Sex Frequency | 41903 | 0.023 ( 0.013, 0.032) | 1.96e-06 | 0.050 ( 0.040, 0.060) | 5.35e-23 | 0.063 ( 0.049, 0.076) | 2.28e-19 |
| Alcohol Frequency | Time to Conception | 34491 | 0.018 (-0.015, 0.050) | 0.291 | 0.030 (-0.005, 0.064) | 0.096 | 0.023 (-0.024, 0.071) | 0.332 |
| Binge Drinking | Age at First Birth | 21114 | -0.085 (-0.156, -0.013) | 0.020 | 0.313 ( 0.256, 0.370) | 7.79e-27 | 0.325 ( 0.267, 0.383) | 2.50e-28 |
| Binge Drinking | Number of Children | 21114 | -0.091 (-0.103, -0.079) | 8.60e-51 | -0.080 (-0.092, -0.068) | 2.38e-36 | -0.082 (-0.095, -0.070) | 1.20e-37 |
| Binge Drinking | Sex Frequency | 20555 | 0.100 ( 0.083, 0.116) | 2.88e-33 | 0.076 ( 0.059, 0.092) | 5.67e-19 | 0.078 ( 0.061, 0.095) | 8.94e-20 |
| Binge Drinking | Time to Conception | 17049 | 0.014 (-0.042, 0.070) | 0.619 | 0.044 (-0.014, 0.103) | 0.134 | 0.046 (-0.012, 0.105) | 0.121 |
| Caffeine Consumption | Age at First Birth | 20227 | 0.215 ( 0.161, 0.269) | 1.01e-14 | -0.098 (-0.142, -0.055) | 9.59e-06 | -0.096 (-0.139, -0.052) | 1.78e-05 |
| Caffeine Consumption | Number of Children | 20227 | 0.011 ( 0.002, 0.020) | 0.019 | 0.007 (-0.003, 0.016) | 0.172 | 0.007 (-0.003, 0.016) | 0.183 |
| Caffeine Consumption | Sex Frequency | 19690 | -0.025 (-0.037, -0.013) | 7.97e-05 | -0.003 (-0.016, 0.009) | 0.597 | -0.003 (-0.016, 0.010) | 0.644 |
| Caffeine Consumption | Time to Conception | 16350 | 0.052 ( 0.010, 0.094) | 0.016 | 0.027 (-0.017, 0.071) | 0.233 | 0.026 (-0.018, 0.070) | 0.250 |
| BMI | Age at First Birth | 45579 | 0.035 ( 0.021, 0.048) | 4.29e-07 | 0.023 ( 0.011, 0.034) | 1.15e-04 | -0.002 (-0.018, 0.013) | 0.761 |
| BMI | Number of Children | 45579 | -0.003 (-0.005, -0.0004) | 0.021 | -0.006 (-0.008, -0.003) | 2.37e-06 | -0.006 (-0.009, -0.003) | 6.26e-04 |
| BMI | Sex Frequency | 44530 | -0.008 (-0.011, -0.005) | 8.28e-08 | -0.008 (-0.011, -0.005) | 1.44e-06 | -0.006 (-0.010, -0.001) | 0.015 |
| BMI | Time to Conception | 36550 | 0.054 ( 0.043, 0.065) | 4.73e-23 | 0.041 ( 0.030, 0.052) | 6.48e-13 | 0.045 ( 0.029, 0.061) | 1.96e-08 |
| Smoking Initiation | Age at First Birth | 44145 | -0.656 (-0.747, -0.565) | 1.68e-45 | -0.201 (-0.280, -0.122) | 5.70e-07 | -0.123 (-0.229, -0.018) | 0.022 |
| Smoking Initiation | Number of Children | 44145 | -0.030 (-0.046, -0.014) | 1.99e-04 | -0.035 (-0.052, -0.018) | 5.54e-05 | -0.029 (-0.052, -0.006) | 0.015 |
| Smoking Initiation | Sex Frequency | 42797 | 0.007 (-0.014, 0.028) | 0.503 | -0.012 (-0.034, 0.009) | 0.268 | 0.013 (-0.018, 0.043) | 0.415 |
| Smoking Initiation | Time to Conception | 35239 | 0.144 ( 0.073, 0.216) | 7.50e-05 | 0.057 (-0.019, 0.132) | 0.141 | -0.014 (-0.120, 0.092) | 0.796 |
| Smoking Heaviness | Age at First Birth | 8255 | -0.030 (-0.048, -0.011) | 0.002 | -0.00030 (-0.017, 0.016) | 0.970 | 0.016 (-0.008, 0.041) | 0.195 |
| Smoking Heaviness | Number of Children | 8255 | -0.004 (-0.007, -0.0006) | 0.022 | -0.005 (-0.008, -0.001) | 0.005 | -0.006 (-0.011, -0.001) | 0.019 |
| Smoking Heaviness | Sex Frequency | 7931 | 0.010 ( 0.005, 0.014) | 6.68e-06 | 0.007 ( 0.003, 0.012) | 0.001 | 0.005 (-0.001, 0.012) | 0.119 |
| Smoking Heaviness | Time to Conception | 6078 | 0.019 ( 0.005, 0.034) | 0.010 | 0.012 (-0.003, 0.028) | 0.125 | 0.007 (-0.017, 0.032) | 0.553 |
| **Exposure** | **Outcome** | **N** | **OR (95% CI)** | **P-value** | **OR (95% CI)** | **P-value** | **OR (95% CI)** | **P-value** |
| Alcohol Frequency | Subfertile | 34491 | 1.020 (0.988, 1.054) | 0.217 | 1.029 (0.994, 1.065) | 0.101 | 1.031 (0.982, 1.082) | 0.223 |
| Alcohol Frequency | Highly Fertile | 42414 | 1.012 (0.992, 1.033) | 0.242 | 1.012 (0.991, 1.035) | 0.268 | 1.018 (0.988, 1.050) | 0.244 |
| Binge Drinking | Subfertile | 17049 | 1.054 (0.995, 1.116) | 0.072 | 1.091 (1.028, 1.158) | 0.004 | 1.090 (1.027, 1.157) | 0.005 |
| Binge Drinking | Highly Fertile | 20795 | 1.022 (0.987, 1.058) | 0.221 | 1.009 (0.972, 1.046) | 0.648 | 1.007 (0.970, 1.045) | 0.722 |
| Caffeine Consumption | Subfertile | 16350 | 1.049 (1.002, 1.098) | 0.041 | 1.028 (0.980, 1.079) | 0.250 | 1.025 (0.977, 1.075) | 0.311 |
| Caffeine Consumption | Highly Fertile | 19926 | 0.990 (0.964, 1.017) | 0.462 | 1.006 (0.978, 1.035) | 0.671 | 1.006 (0.978, 1.035) | 0.666 |
| BMI | Subfertile | 36550 | 1.043 (1.033, 1.054) | 2.02e-16 | 1.035 (1.023, 1.046) | 7.59e-10 | 1.044 (1.028, 1.060) | 4.41e-08 |
| BMI | Highly Fertile | 45051 | 0.976 (0.969, 0.982) | 9.91e-13 | 0.981 (0.974, 0.988) | 8.06e-08 | 0.986 (0.976, 0.996) | 0.006 |
| Smoking Initiation | Subfertile | 35239 | 1.088 (1.014, 1.168) | 0.019 | 1.009 (0.936, 1.088) | 0.813 | 0.989 (0.886, 1.105) | 0.849 |
| Smoking Initiation | Highly Fertile | 43327 | 0.956 (0.914, 0.999) | 0.044 | 0.986 (0.941, 1.034) | 0.569 | 1.028 (0.961, 1.101) | 0.419 |
| Smoking Heaviness | Subfertile | 6078 | 1.018 (1.006, 1.031) | 0.004 | 1.012 (0.999, 1.026) | 0.072 | 1.008 (0.986, 1.030) | 0.469 |
| Smoking Heaviness | Highly Fertile | 8076 | 1.000 (0.992, 1.009) | 0.915 | 1.005 (0.996, 1.014) | 0.284 | 1.004 (0.990, 1.019) | 0.553 |

**Supplementary Table S11. Individual-level Mendelian randomisation analysis in females**

| **Exposure** | **Outcome** | **N** | **Beta/RD (95% CI)** | **P-value** |
| --- | --- | --- | --- | --- |
| Alcohol Consumption | Age at First Birth | 61,599 | 0.109 (-0.683, 0.900) | 0.788 |
|  | Number of Children | 61,599 | -0.131 (-0.309, 0.046) | 0.147 |
|  | Sex Frequency | 60,464 | 0.149 (-0.080, 0.378) | 0.202 |
|  | Time to Conception | 48,532 | 0.300 (-0.491, 1.091) | 0.457 |
|  | Subfertile | 48,532 | 0.021 (-0.047, 0.090) | 0.544 |
|  | Highly Fertile | 60,707 | -0.035 (-0.123, 0.054) | 0.442 |
|  | Infertility Treatment | 60,970 | 0.002 (-0.059, 0.062) | 0.953 |
|  | Miscarriage | 41,634 | -0.002 (-0.111, 0.107) | 0.973 |
| Caffeine Consumption | Age at First Birth | 56,874 | 0.372 (-0.082, 0.826) | 0.109 |
|  | Number of Children | 56,874 | -0.058 (-0.159, 0.043) | 0.262 |
|  | Sex Frequency | 55,840 | -0.020 (-0.148, 0.109) | 0.765 |
|  | Time to Conception | 44,628 | 0.290 (-0.162, 0.742) | 0.209 |
|  | Subfertile | 44,628 | 0.041 ( 0.001, 0.080) | 0.044 |
|  | Highly Fertile | 56,059 | 0.024 (-0.026, 0.074) | 0.350 |
|  | Infertility Treatment | 56,235 | -0.002 (-0.036, 0.032) | 0.890 |
|  | Miscarriage | 38,707 | -0.003 (-0.070, 0.065) | 0.941 |
| BMI | Age at First Birth | 62,545 | -0.162 (-0.189, -0.136) | 2.45e-32 |
|  | Number of Children | 62,545 | 0.002 (-0.004, 0.008) | 0.559 |
|  | Sex Frequency | 61,657 | 0.012 ( 0.004, 0.020) | 0.003 |
|  | Time to Conception | 49,310 | 0.049 ( 0.020, 0.077) | 7.57e-04 |
|  | Subfertile | 49,310 | 0.006 ( 0.003, 0.008) | 2.82e-06 |
|  | Highly Fertile | 61,905 | -0.0002 (-0.003, 0.003) | 0.888 |
|  | Infertility Treatment | 62,111 | 0.002 (-0.0003, 0.004) | 0.089 |
|  | Miscarriage | 42,436 | -0.004 (-0.008, 0.0001) | 0.058 |
| Smoking Initiation | Age at First Birth | 63,376 | -3.344 (-3.890, -2.797) | 4.44e-33 |
|  | Number of Children | 63,376 | 0.012 (-0.106, 0.129) | 0.843 |
|  | Sex Frequency | 62,176 | -0.012 (-0.163, 0.138) | 0.871 |
|  | Time to Conception | 49,925 | 0.462 (-0.054, 0.978) | 0.080 |
|  | Subfertile | 49,925 | 0.036 (-0.009, 0.081) | 0.114 |
|  | Highly Fertile | 62,439 | -0.059 (-0.118, -0.0008) | 0.047 |
|  | Infertility Treatment | 62,717 | 0.029 (-0.011, 0.068) | 0.153 |
|  | Miscarriage | 42,863 | -0.068 (-0.142, 0.007) | 0.075 |
| Smoking Heaviness | Age at First Birth | 12,140 | 0.039 (-0.085, 0.162) | 0.538 |
|  | Number of Children | 12,140 | 0.005 (-0.022, 0.032) | 0.707 |
|  | Sex Frequency | 11,892 | -0.0009 (-0.037, 0.035) | 0.962 |
|  | Time to Conception | 8,374 | 0.046 (-0.084, 0.176) | 0.491 |
|  | Subfertile | 8,374 | -0.002 (-0.014, 0.009) | 0.702 |
|  | Highly Fertile | 11,894 | -0.007 (-0.019, 0.005) | 0.260 |
|  | Infertility Treatment | 11,990 | -0.0010 (-0.009, 0.007) | 0.806 |
|  | Miscarriage | 8,074 | -0.004 (-0.021, 0.012) | 0.613 |

**Supplementary Table S12. Individual-level Mendelian randomisation analysis in males**

| **Exposure** | **Outcome** | **N** | **Beta/RD (95% CI)** | **P-value** |
| --- | --- | --- | --- | --- |
| Alcohol Consumption | Age at First Birth | 43,092 | -1.024 (-1.785, -0.263) | 0.008 |
|  | Number of Children | 43,092 | 0.053 (-0.097, 0.203) | 0.489 |
|  | Sex Frequency | 41,760 | 0.162 (-0.036, 0.360) | 0.109 |
|  | Time to Conception | 34,378 | 0.802 ( 0.097, 1.507) | 0.026 |
|  | Subfertile | 34,378 | 0.055 (-0.005, 0.115) | 0.075 |
|  | Highly Fertile | 42,273 | -0.037 (-0.114, 0.040) | 0.342 |
| Caffeine Consumption | Age at First Birth | 20,154 | 0.570 (-0.357, 1.496) | 0.228 |
|  | Number of Children | 20,154 | -0.012 (-0.207, 0.183) | 0.904 |
|  | Sex Frequency | 19,604 | -0.151 (-0.416, 0.114) | 0.265 |
|  | Time to Conception | 16,279 | -0.405 (-1.257, 0.448) | 0.352 |
|  | Subfertile | 16,279 | -0.072 (-0.148, 0.004) | 0.064 |
|  | Highly Fertile | 19,841 | -0.042 (-0.145, 0.061) | 0.422 |
| BMI | Age at First Birth | 45,460 | -0.145 (-0.189, -0.100) | 1.68e-10 |
|  | Number of Children | 45,460 | 0.0008 (-0.008, 0.010) | 0.867 |
|  | Sex Frequency | 44,383 | -0.006 (-0.018, 0.006) | 0.297 |
|  | Time to Conception | 36,433 | 0.037 (-0.005, 0.079) | 0.085 |
|  | Subfertile | 36,433 | 0.003 (-0.0002, 0.007) | 0.068 |
|  | Highly Fertile | 44,906 | -0.005 (-0.010, -0.0005) | 0.030 |
| Smoking Initiation | Age at First Birth | 44,025 | -2.478 (-3.164, -1.792) | 1.45e-12 |
|  | Number of Children | 44,025 | -0.016 (-0.157, 0.124) | 0.822 |
|  | Sex Frequency | 42,651 | 0.004 (-0.179, 0.187) | 0.963 |
|  | Time to Conception | 35,123 | 0.818 ( 0.186, 1.451) | 0.011 |
|  | Subfertile | 35,123 | 0.033 (-0.021, 0.088) | 0.232 |
|  | Highly Fertile | 43,183 | -0.097 (-0.168, -0.025) | 0.008 |
| Smoking Heaviness | Age at First Birth | 8,191 | -0.029 (-0.197, 0.139) | 0.736 |
|  | Number of Children | 8,191 | -0.021 (-0.056, 0.014) | 0.240 |
|  | Sex Frequency | 7,861 | 0.011 (-0.035, 0.056) | 0.647 |
|  | Time to Conception | 6,019 | 0.023 (-0.129, 0.175) | 0.767 |
|  | Subfertile | 6,019 | 0.005 (-0.008, 0.019) | 0.443 |
|  | Highly Fertile | 8,005 | 0.006 (-0.011, 0.022) | 0.500 |

**Supplementary Table S13. Evidence for assortative mating: the association of PGS on partners health behaviours and correlation between PGS**

| **PGS** | **Exposure in Partner** | **N** | **Beta/OR (95% CI)** | **P-value** |
| --- | --- | --- | --- | --- |
| Female DPW p<5x10-8 | Male Alcohol Frequency | 48,359 | 0.016 ( 0.006, 0.026) | 0.001 |
| Male DPW p<5x10-8 | Female Alcohol Frequency | 44,909 | 0.017 ( 0.007, 0.027) | 8.00e-04 |
| Female Caffeine p<5x10-6 | Male Caffeine Consumption | 21,040 | 0.005 (-0.012, 0.021) | 0.572 |
| Male Caffeine p<5x10-6 | Female Caffeine Consumption | 40,983 | 0.006 (-0.005, 0.018) | 0.295 |
| Female BMI p<5x10-8 | Male BMI | 63,114 | 0.161 ( 0.135, 0.186) | 3.27e-34 |
| Male BMI p<5x10-8 | Female BMI | 45,507 | 0.205 ( 0.166, 0.244) | 1.84e-24 |
| Female SI p<5x10-8 | Male Smoking Initiation | 49,444 | 1.088 ( 1.069, 1.108) | 9.22e-21 |
| Male SI p<5x10-8 | Female Smoking Initiation | 46,269 | 1.105 ( 1.085, 1.126) | 8.94e-27 |
| Female CPD p<5x10-8 | Male Smoking Heaviness | 9,388 | 0.143 ( 0.016, 0.270) | 0.028 |
| Male CPD p<5x10-8 | Female Smoking Heaviness | 8,113 | 0.119 (-0.009, 0.246) | 0.068 |
| **PGS** | **PGS** | **N** | **r** | **P-value** |
| Female DPW p<5x10-8 | Male DPW p<5x10-8 | 37,565 | 0.013 | 0.01 |
| Female Caffeine p<5x10-6 | Male Caffeine p<5x10-6 | 37,565 | -0.002 | 0.72 |
| Female BMI p<5x10-8 | Male BMI p<5x10-8 | 37,565 | 0.008 | 0.11 |
| Female SI p<5x10-8 | Male SI p<5x10-8 | 37,565 | 0.008 | 0.10 |
| Female CPD p<5x10-8 | Male CPD p<5x10-8 | 37,565 | 0.009 | 0.08 |

**Supplementary Table S14. Evidence for reintroduced confounding: associations between PGS and other health behaviours or confounders**

| **PRS** | **Confounder** | **N** | **Beta/OR (95% CI)** | **P-value** |
| --- | --- | --- | --- | --- |
| Mother DPW p<5x10-8 | Age | 70,856 | -0.028 (-0.062, 0.006) | 0.109 |
| Mother DPW p<5x10-8 | Income | 60,885 | -0.017 (-0.035, 0.0002) | 0.053 |
| Mother DPW p<5x10-8 | Binge Drinking | 61,134 | 0.034 ( 0.026, 0.041) | 6.88e-20 |
| Mother DPW p<5x10-8 | Caffeine Consumption | 57,911 | 0.030 ( 0.020, 0.040) | 2.87e-09 |
| Mother DPW p<5x10-8 | BMI | 63,658 | -0.002 (-0.035, 0.031) | 0.906 |
| Mother DPW p<5x10-8 | Smoking Heaviness | 12,615 | 0.061 (-0.042, 0.165) | 0.246 |
| Mother Caffeine p<5x10-8 | Age | 70,856 | -0.015 (-0.049, 0.019) | 0.384 |
| Mother Caffeine p<5x10-8 | Income | 60,885 | 0.019 ( 0.001, 0.037) | 0.034 |
| Mother Caffeine p<5x10-8 | Alcohol Frequency | 61,750 | 0.004 (-0.005, 0.012) | 0.420 |
| Mother Caffeine p<5x10-8 | Binge Drinking | 61,134 | 0.0006 (-0.007, 0.008) | 0.861 |
| Mother Caffeine p<5x10-8 | BMI | 63,658 | -0.009 (-0.043, 0.024) | 0.596 |
| Mother Caffeine p<5x10-8 | Smoking Heaviness | 12,615 | 0.033 (-0.070, 0.137) | 0.527 |
| Mother BMI p<5x10-8 | Age | 70,856 | -0.181 (-0.215, -0.146) | 4.01e-25 |
| Mother BMI p<5x10-8 | Income | 60,885 | -0.097 (-0.115, -0.080) | 2.67e-27 |
| Mother BMI p<5x10-8 | Alcohol Frequency | 61,750 | -0.040 (-0.049, -0.032) | 4.43e-20 |
| Mother BMI p<5x10-8 | Binge Drinking | 61,134 | 0.009 ( 0.002, 0.017) | 0.010 |
| Mother BMI p<5x10-8 | Caffeine Consumption | 57,911 | 0.038 ( 0.028, 0.048) | 3.09e-14 |
| Mother BMI p<5x10-8 | Smoking Heaviness | 12,615 | 0.361 ( 0.258, 0.464) | 7.12e-12 |
| Mother SI p<5x10-8 | Age | 70,856 | -0.175 (-0.209, -0.140) | 1.29e-23 |
| Mother SI p<5x10-8 | Income | 60,885 | -0.101 (-0.119, -0.084) | 2.45e-29 |
| Mother SI p<5x10-8 | Alcohol Frequency | 61,750 | 0.005 (-0.004, 0.013) | 0.305 |
| Mother SI p<5x10-8 | Binge Drinking | 61,134 | 0.050 ( 0.043, 0.058) | 1.12e-42 |
| Mother SI p<5x10-8 | Caffeine Consumption | 57,911 | 0.071 ( 0.061, 0.081) | 1.73e-45 |
| Mother SI p<5x10-8 | BMI | 63,658 | 0.158 ( 0.125, 0.192) | 1.51e-20 |
| Mother SI p<5x10-8 | Smoking Heaviness | 12,615 | 0.270 ( 0.165, 0.375) | 4.43e-07 |
| Mother CPD p<5x10-8 | Age | 70,856 | -0.023 (-0.057, 0.011) | 0.188 |
| Mother CPD p<5x10-8 | Income | 60,885 | -0.008 (-0.026, 0.009) | 0.351 |
| Mother CPD p<5x10-8 | Alcohol Frequency | 61,750 | -0.014 (-0.023, -0.006) | 0.001 |
| Mother CPD p<5x10-8 | Binge Drinking | 61,134 | -0.002 (-0.009, 0.006) | 0.644 |
| Mother CPD p<5x10-8 | Caffeine Consumption | 57,911 | 0.012 ( 0.002, 0.022) | 0.020 |
| Mother CPD p<5x10-8 | BMI | 63,658 | 0.059 ( 0.025, 0.092) | 5.64e-04 |
| Father DPW p<5x10-8 | Age | 70,856 | -0.028 (-0.062, 0.006) | 0.109 |
| Father DPW p<5x10-8 | Income | 60,885 | -0.017 (-0.035, 0.0002) | 0.053 |
| Father DPW p<5x10-8 | Binge Drinking | 61,134 | 0.034 ( 0.026, 0.041) | 6.88e-20 |
| Father DPW p<5x10-8 | Caffeine Consumption | 57,911 | 0.030 ( 0.020, 0.040) | 2.87e-09 |
| Father DPW p<5x10-8 | BMI | 63,658 | -0.002 (-0.035, 0.031) | 0.906 |
| Father DPW p<5x10-8 | Smoking Heaviness | 12,615 | 0.061 (-0.042, 0.165) | 0.246 |
| Father Caffeine p<5x10-8 | Age | 70,856 | -0.015 (-0.049, 0.019) | 0.384 |
| Father Caffeine p<5x10-8 | Income | 60,885 | 0.019 ( 0.001, 0.037) | 0.034 |
| Father Caffeine p<5x10-8 | Alcohol Frequency | 61,750 | 0.004 (-0.005, 0.012) | 0.420 |
| Father Caffeine p<5x10-8 | Binge Drinking | 61,134 | 0.0006 (-0.007, 0.008) | 0.861 |
| Father Caffeine p<5x10-8 | BMI | 63,658 | -0.009 (-0.043, 0.024) | 0.596 |
| Father Caffeine p<5x10-8 | Smoking Heaviness | 12,615 | 0.033 (-0.070, 0.137) | 0.527 |
| Father BMI p<5x10-8 | Age | 70,856 | -0.181 (-0.215, -0.146) | 4.01e-25 |
| Father BMI p<5x10-8 | Income | 60,885 | -0.097 (-0.115, -0.080) | 2.67e-27 |
| Father BMI p<5x10-8 | Alcohol Frequency | 61,750 | -0.040 (-0.049, -0.032) | 4.43e-20 |
| Father BMI p<5x10-8 | Binge Drinking | 61,134 | 0.009 ( 0.002, 0.017) | 0.010 |
| Father BMI p<5x10-8 | Caffeine Consumption | 57,911 | 0.038 ( 0.028, 0.048) | 3.09e-14 |
| Father BMI p<5x10-8 | Smoking Heaviness | 12,615 | 0.361 ( 0.258, 0.464) | 7.12e-12 |
| Father SI p<5x10-8 | Age | 70,856 | -0.175 (-0.209, -0.140) | 1.29e-23 |
| Father SI p<5x10-8 | Income | 60,885 | -0.101 (-0.119, -0.084) | 2.45e-29 |
| Father SI p<5x10-8 | Alcohol Frequency | 61,750 | 0.005 (-0.004, 0.013) | 0.305 |
| Father SI p<5x10-8 | Binge Drinking | 61,134 | 0.050 ( 0.043, 0.058) | 1.12e-42 |
| Father SI p<5x10-8 | Caffeine Consumption | 57,911 | 0.071 ( 0.061, 0.081) | 1.73e-45 |
| Father SI p<5x10-8 | BMI | 63,658 | 0.158 ( 0.125, 0.192) | 1.51e-20 |
| Father SI p<5x10-8 | Smoking Heaviness | 12,615 | 0.270 ( 0.165, 0.375) | 4.43e-07 |
| Father CPD p<5x10-8 | Age | 70,856 | -0.023 (-0.057, 0.011) | 0.188 |
| Father CPD p<5x10-8 | Income | 60,885 | -0.008 (-0.026, 0.009) | 0.351 |
| Father CPD p<5x10-8 | Alcohol Frequency | 61,750 | -0.014 (-0.023, -0.006) | 0.001 |
| Father CPD p<5x10-8 | Binge Drinking | 61,134 | -0.002 (-0.009, 0.006) | 0.644 |
| Father CPD p<5x10-8 | Caffeine Consumption | 57,911 | 0.012 ( 0.002, 0.022) | 0.020 |
| Father CPD p<5x10-8 | BMI | 63,658 | 0.059 ( 0.025, 0.092) | 5.64e-04 |

**Supplementary Table S15. Summary level MR sensitivity tests conducted in the MoBa sample**

|  |  |  | Females |  | Males |  |
| --- | --- | --- | --- | --- | --- | --- |
| Exposure | **Outcome** | **Method** | **Beta (95% CI)** | **P-value** | **Beta (95% CI)** | **P-value** |
| BMI | Age at first birth | IVW | -0.536 (-0.651, -0.422) | <0.001 | -0.386 (-0.523, -0.25) | <0.001 |
|  |  | MR Egger | -0.315 (-0.638, 0.008) | 0.06 | -0.485 (-0.87, -0.099) | 0.01 |
|  |  | *MR Egger Intercept* | *-0.003 (-0.008, 0.001)* | *0.15* | *0.002 (-0.004, 0.007)* | *0.59* |
|  |  | Weighted Median | -0.418 (-0.587, -0.25) | <0.001 | -0.355 (-0.576, -0.134) | 0.002 |
|  |  | Weighted Mode | -0.283 (-0.677, 0.11) | 0.16 | -0.372 (-0.984, 0.239) | 0.23 |
| BMI | Time to conception | IVW | 0.202 (0.092, 0.313) | <0.001 | - | - |
|  |  | MR Egger | 0.245 (-0.068, 0.558) | 0.13 | - | - |
|  |  | *MR Egger Intercept* | *-0.001 (-0.005, 0.004)* | *0.77* | - | - |
|  |  | Weighted Median | 0.065 (-0.12, 0.251) | 0.49 | - | - |
|  |  | Weighted Mode | -0.052 (-0.424, 0.32) | 0.79 | - | - |
| Smoking Initiation | Age at first birth | IVW | -0.784 (-0.998, -0.57) | <0.001 | -0.662 (-0.901, -0.422) | <0.001 |
|  |  | MR Egger | -0.961 (-1.859, -0.063) | 0.04 | -1.000 (-2.006, 0.005) | 0.05 |
|  |  | *MR Egger Intercept* | *0.003 (-0.014, 0.021)* | *0.69* | *0.007 (-0.013, 0.026)* | *0.50* |
|  |  | Weighted Median | -0.773 (-1.026, -0.52) | <0.001 | -0.764 (-1.088, -0.44) | <0.001 |
|  |  | Weighted Mode | -0.787 (-1.516, -0.058) | 0.04 | -0.72 (-1.517, 0.077) | 0.08 |
| Exposure | **Outcome** | **Method** | **Odds Ratio (95% CI)** | **P-value** |  |  |
| BMI | Subfertility | IVW | 1.224 (1.096, 1.368) | <0.001 | - | - |
|  |  | MR Egger | 1.278 (0.934, 1.748) | 0.13 | - | - |
|  |  | *MR Egger Intercept* | *-0.001 (-0.005, 0.004)* | *0.77* | *-* | *-* |
|  |  | Weighted Median | 1.067 (0.879, 1.296) | 0.51 | - | - |
|  |  | Weighted Mode | 0.95 (0.661, 1.364) | 0.78 | - | - |

**Supplementary Table S16. Comparison of individual-level MR results in females between first pregnancy and full sample**

| **Exposure** | **Pregnancy** | **Outcome** | **N** | **Beta/RD (95% CI)** | **P-value** |
| --- | --- | --- | --- | --- | --- |
| Alcohol Consumption | Full Sample | Number of Children | 61,599 | -0.131 (-0.309, 0.046) | 0.147 |
|  | 1st Pregnancy | Number of Children | 19,176 | -0.345 (-0.688, -0.0008) | 0.049 |
|  | Full Sample | Age at First Birth | 61,599 | 0.109 (-0.683, 0.900) | 0.788 |
|  | 1st Pregnancy | Age at First Birth | 19,176 | -0.270 (-1.054, 0.514) | 0.499 |
|  | Full Sample | Time to Conception | 48,532 | 0.300 (-0.491, 1.091) | 0.457 |
|  | 1st Pregnancy | Time to Conception | 14,539 | -0.791 (-2.528, 0.946) | 0.372 |
|  | Full Sample | Infertility Treatment | 60,970 | 0.002 (-0.059, 0.062) | 0.953 |
|  | 1st Pregnancy | Infertility Treatment | 19,062 | -0.070 (-0.193, 0.052) | 0.262 |
| BMI | Full Sample | Number of Children | 62,545 | 0.002 (-0.004, 0.008) | 0.559 |
|  | 1st Pregnancy | Number of Children | 19,507 | -0.023 (-0.032, -0.014) | 1.13e-06 |
|  | Full Sample | Age at First Birth | 62,545 | -0.162 (-0.189, -0.136) | 2.45e-32 |
|  | 1st Pregnancy | Age at First Birth | 19,507 | -0.051 (-0.074, -0.028) | 1.05e-05 |
|  | Full Sample | Time to Conception | 49,310 | 0.049 ( 0.020, 0.077) | 7.57e-04 |
|  | 1st Pregnancy | Time to Conception | 14,820 | 0.057 ( 0.001, 0.113) | 0.045 |
|  | Full Sample | Infertility Treatment | 62,111 | 0.002 (-0.0003, 0.004) | 0.089 |
|  | 1st Pregnancy | Infertility Treatment | 19,395 | 0.004 ( 0.0002, 0.007) | 0.038 |
| Caffeine Consumption | Full Sample | Number of Children | 56,874 | -0.058 (-0.159, 0.043) | 0.262 |
|  | 1st Pregnancy | Number of Children | 17,393 | 0.030 (-0.111, 0.172) | 0.673 |
|  | Full Sample | Age at First Birth | 56,874 | 0.372 (-0.082, 0.826) | 0.109 |
|  | 1st Pregnancy | Age at First Birth | 17,393 | 0.328 (-0.021, 0.677) | 0.066 |
|  | Full Sample | Time to Conception | 44,628 | 0.290 (-0.162, 0.742) | 0.209 |
|  | 1st Pregnancy | Time to Conception | 13,206 | 0.325 (-0.521, 1.171) | 0.451 |
|  | Full Sample | Infertility Treatment | 56,235 | -0.002 (-0.036, 0.032) | 0.890 |
|  | 1st Pregnancy | Infertility Treatment | 17,290 | 0.0005 (-0.055, 0.056) | 0.986 |
| Smoking Heaviness | Full Sample | Number of Children | 12,140 | 0.005 (-0.022, 0.032) | 0.707 |
|  | 1st Pregnancy | Number of Children | 3,818 | 0.011 (-0.028, 0.050) | 0.568 |
|  | Full Sample | Age at First Birth | 12,140 | 0.039 (-0.085, 0.162) | 0.538 |
|  | 1st Pregnancy | Age at First Birth | 3,818 | 0.024 (-0.070, 0.118) | 0.619 |
|  | Full Sample | Time to Conception | 8,374 | 0.046 (-0.084, 0.176) | 0.491 |
|  | 1st Pregnancy | Time to Conception | 2,423 | -0.133 (-0.374, 0.108) | 0.280 |
|  | Full Sample | Infertility Treatment | 11,990 | -0.0010 (-0.009, 0.007) | 0.806 |
|  | 1st Pregnancy | Infertility Treatment | 3,788 | -0.009 (-0.022, 0.004) | 0.176 |
| Smoking Initiation | Full Sample | Number of Children | 63,376 | 0.012 (-0.106, 0.129) | 0.843 |
|  | 1st Pregnancy | Number of Children | 19,709 | -0.327 (-0.515, -0.139) | 6.70e-04 |
|  | Full Sample | Age at First Birth | 63,376 | -3.344 (-3.890, -2.797) | 4.44e-33 |
|  | 1st Pregnancy | Age at First Birth | 19,709 | -1.191 (-1.664, -0.718) | 8.00e-07 |
|  | Full Sample | Time to Conception | 49,925 | 0.462 (-0.054, 0.978) | 0.080 |
|  | 1st Pregnancy | Time to Conception | 14,947 | 0.266 (-0.777, 1.309) | 0.617 |
|  | Full Sample | Infertility Treatment | 62,717 | 0.029 (-0.011, 0.068) | 0.153 |
|  | 1st Pregnancy | Infertility Treatment | 19,589 | 0.028 (-0.044, 0.101) | 0.442 |

**Supplementary Table S17. Comparison of individual-level MR results in males between first pregnancy and full sample**

| **Exposure** | **Pregnancy** | **Outcome** | **N** | **Beta/RD (95% CI)** | **P-value** |
| --- | --- | --- | --- | --- | --- |
| Alcohol Consumption | Full Sample | Number of Children | 43,092 | 0.053 (-0.097, 0.203) | 0.489 |
|  | Partner's 1st Pregnancy | Number of Children | 14,348 | 0.104 (-0.189, 0.397) | 0.486 |
|  | Full Sample | Age at First Birth | 43,092 | -1.024 (-1.785, -0.263) | 0.008 |
|  | Partner's 1st Pregnancy | Age at First Birth | 14,348 | -0.800 (-2.189, 0.589) | 0.259 |
|  | Full Sample | Time to Conception | 34,378 | 0.802 ( 0.097, 1.507) | 0.026 |
|  | Partner's 1st Pregnancy | Time to Conception | 11,197 | 0.895 (-0.691, 2.481) | 0.269 |
| BMI | Full Sample | Number of Children | 45,460 | 0.0008 (-0.008, 0.010) | 0.867 |
|  | Partner's 1st Pregnancy | Number of Children | 15,206 | -0.008 (-0.022, 0.006) | 0.267 |
|  | Full Sample | Age at First Birth | 45,460 | -0.145 (-0.189, -0.100) | 1.68e-10 |
|  | Partner's 1st Pregnancy | Age at First Birth | 15,206 | -0.102 (-0.165, -0.040) | 0.001 |
|  | Full Sample | Time to Conception | 36,433 | 0.037 (-0.005, 0.079) | 0.085 |
|  | Partner's 1st Pregnancy | Time to Conception | 11,856 | 0.074 ( 0.002, 0.146) | 0.044 |
| Caffeine Consumption | Full Sample | Number of Children | 20,154 | -0.012 (-0.207, 0.183) | 0.904 |
|  | Partner's 1st Pregnancy | Number of Children | 7,506 | 0.069 (-0.281, 0.419) | 0.697 |
|  | Full Sample | Age at First Birth | 20,154 | 0.570 (-0.357, 1.496) | 0.228 |
|  | Partner's 1st Pregnancy | Age at First Birth | 7,506 | 0.620 (-0.936, 2.177) | 0.435 |
|  | Full Sample | Time to Conception | 16,279 | -0.405 (-1.257, 0.448) | 0.352 |
|  | Partner's 1st Pregnancy | Time to Conception | 5,890 | -0.709 (-2.778, 1.360) | 0.502 |
| Smoking Heaviness | Full Sample | Number of Children | 8,191 | -0.021 (-0.056, 0.014) | 0.240 |
|  | Partner's 1st Pregnancy | Number of Children | 2,690 | 0.0001 (-0.063, 0.064) | 0.997 |
|  | Full Sample | Age at First Birth | 8,191 | -0.029 (-0.197, 0.139) | 0.736 |
|  | Partner's 1st Pregnancy | Age at First Birth | 2,690 | 0.120 (-0.181, 0.421) | 0.436 |
|  | Full Sample | Time to Conception | 6,019 | 0.023 (-0.129, 0.175) | 0.767 |
|  | Partner's 1st Pregnancy | Time to Conception | 1,884 | -0.012 (-0.317, 0.293) | 0.939 |
| Smoking Initiation | Full Sample | Number of Children | 44,025 | -0.016 (-0.157, 0.124) | 0.822 |
|  | Partner's 1st Pregnancy | Number of Children | 14,671 | 0.114 (-0.127, 0.356) | 0.354 |
|  | Full Sample | Age at First Birth | 44,025 | -2.478 (-3.164, -1.792) | 1.45e-12 |
|  | Partner's 1st Pregnancy | Age at First Birth | 14,671 | -2.803 (-3.940, -1.666) | 1.36e-06 |
|  | Full Sample | Time to Conception | 35,123 | 0.818 ( 0.186, 1.451) | 0.011 |
|  | Partner's 1st Pregnancy | Time to Conception | 11,453 | 0.475 (-0.697, 1.646) | 0.427 |

**Supplementary Table S18. Evidence for heterogeneity: Cochran’s Q statistics**

| Exposure | Outcome | Q | df | P Value |
| --- | --- | --- | --- | --- |
| BMI | Age at First Birth | 268.13 | 94 | 1.18 x 10^-18^ |
|  | Number of Children | 200.16 | 90 | 2.35 x 10^-10^ |
|  | Number of miscarriages | 121.15 | 94 | 0.03 |
| Alcohol Frequency | Age at First Birth | 254.81 | 93 | 5.44 x 10^-17^ |
|  | Number of Children | 224.34 | 82 | 3.60 x 10^-15^ |
|  | Number of miscarriages | 128.72 | 93 | 0.008 |
| Smoking Initiation | Age at First Birth | 1052.53 | 321 | 1.16 x -78 |
|  | Number of Children | 871.59 | 322 | 3.52 x 10^-52^ |
|  | Number of miscarriages | 356.24 | 321 | 0.09 |
| Caffeine Consumption | Age at First Birth | 12.25 | 5 | 0.03 |
|  | Number of Children | 2.74 | 5 | 0.74 |
|  | Number of miscarriages | 1.87 | 5 | 0.87 |

**Supplementary Table S19. The MR Egger intercept test: Evidence for bias from horizontal pleiotropy**

| Exposure | Outcome | Intercept (95% CI) | P Value |
| --- | --- | --- | --- |
| BMI | Age at first birth | -0.001 (-0.005, 0.003) | 0.65 |
|  | Number of Children | -0.0003 (-0.003, 0.003) | 0.83 |
|  | Number of miscarriages | -0.0004 (-0.003, 0.002) | 0.74 |
| Alcohol Frequency | Age at first birth | -0.002 (-0.004, 0) | 0.07 |
|  | Number of Children | 0.0007 (-0.001, 0.003) | 0.49 |
|  | Number of miscarriages | -0.0002 (-0.002, 0.001) | 0.78 |
| Smoking Initiation | Age at first birth | -0.001 (-0.003, 0.001) | 0.15 |
|  | Number of Children | 0.001 (-0.0003, 0.003) | 0.11 |
|  | Number of miscarriages | -0.0002 (-0.001, 0.001) | 0.79 |
| Caffeine Consumption | Age at first birth | -0.02 (-0.036, -0.004) | 0.07 |
|  | Number of children | 0.0001 (-0.012, 0.013) | 0.98 |
|  | Number of miscarriages | 0.006 (-0.01, 0.021) | 0.52 |

**Supplementary Table S20. Steiger filtering test for possible reverse causation**

| Exposure | Outcome | N SNPs | N After Steiger Filtering | % True |
| --- | --- | --- | --- | --- |
| BMI | Age at First Birth | 96 | 96 | 100 |
|  | Number of Children | 91 | 91 | 100 |
|  | Number of miscarriages | 96 | 96 | 100 |
| Alcohol Frequency | Age at First Birth | 96 | 80 | 83 |
|  | Number of Children | 83 | 83 | 100 |
|  | Number of miscarriages | 96 | 87 | 91 |
| Smoking Initiation | Age at First Birth | 368 | 267 | 73 |
|  | Number of Children | 323 | 278 | 86 |
|  | Number of miscarriages | 368 | 352 | 96 |
| Caffeine Consumption | Age at First Birth | 6 | 6 | 100 |
|  | Number of Children | 6 | 6 | 100 |
|  | Number of miscarriages | 6 | 6 | 100 |

**Supplementary Table S21. Test of instrument strength and the suitability of the instrument for MR Egger**

| Exposure | Outcome | mF | Unweighted I^2^_GX_ | Weighted I^2^_GX_ |
| --- | --- | --- | --- | --- |
| BMI | Age at First Birth | 59.87 | 0.904 | 0.889 |
|  | Number of Children | 59.87 | 0.904 | 0.889 |
|  | Number of miscarriages | 59.87 | 0.904 | 0.889 |
| Alcohol Frequency | Age at First Birth | 14.87 | 0.827 | 0.790 |
|  | Number of Children | 14.72 | 0.825 | 0.832 |
|  | Number of miscarriages | 14.87 | 0.827 | 0.792 |
| Smoking Initiation | Age at First Birth | 7.72 | 0.08 | 0 |
|  | Number of Children | 7.70 | 0.077 | 0.046 |
|  | Number of miscarriages | 7.72 | 0.08 | 0 |
| Caffeine Consumption | Age at First Birth | 27.83 | 0.268 | 0.172 |
|  | Number of Children | 27.83 | 0.268 | 0.177 |
|  | Number of miscarriages | 27.83 | 0.268 | 0.175 |

**Supplementary Table S22. Exploratory multivariable Mendelian randomisation estimating the direct effects of smoking initiation and BMI on age at first birth, accounting for education and impulsivity**

| Exposure |  | mF | N SNP | Beta (95% CI) | P Value |
| --- | --- | --- | --- | --- | --- |
| Educational attainment^a^ | Univariable MR | 88.67 | 67 | 0.595 (0.518, 0.672) | 2.40 x 10^-51^ |
|  | Multivariable MR accounting for SI | 5.28 | 182 | 0.621 (0.528, 0.714) | <2.2 x 10^-16^ |
|  | Multivariable MR accounting for BMI | 11.91 | 88 | 0.639 (0.514, 0.764) | 3.59 x 10^-16^ |
| ADHD^b^ | Univariable MR | 13.67 | 9 | -0.108 (-0.150, -0.067) | 2.69 x 10^-7^ |
|  | Multivariable MR accounting for SI | 2.36 | 180 | -0.096 (-0.133, -0.059) | 1.74 x 10^-6^ |
|  | Multivariable MR accounting for BMI | 4.82 | 78 | -0.109 (-0.147, -0.071) | 2.67 x 10^-7^ |
| Smoking initiation | Univariable MR | 7.70 | 352 | -0.272 (-0.31, -0.234) | 3.45 x 10^-45^ |
|  | Multivariable MR accounting for ADHD | 3.09 | 180 | -0.435 (-0.591, -0.279) | 1.70 x 10^-7^ |
|  | Multivariable MR accounting for educational attainment | 4.23 | 182 | -0.403 (-0.527, -0.279) | 1.43 x 10^-9^ |
| Body Mass Index | Univariable MR | 4.76 | 94 | -0.112 (-0.163, -0.061) | 1.66 x 10^-5^ |
|  | Multivariable MR accounting for ADHD | 24.19 | 78 | -0.513 (-0.106, 0.003) | 0.07 |
|  | Multivariable MR accounting for educational attainment | 29.65 | 88 | -0.056 (-0.113, 0.0008) | 0.06 |

Note. a = educational attainment was measured as years in education. There were 74 independent genome-wide significant SNPs (Okbay et al., 2016).

b = attention deficit hyperactivity disorder (ADHD) was used as a proxy for impulsivity (Demontis et al., 2019). There were 9 SNPs independently associated with ADHD at p<5x10-6.

**References**

References included in the main manuscript.
